# Taxonomy and systematics of *Emprostiotrema* Cianferoni and Ceccolini, 2021 (Digenea: Emprostiotrematidae), parasites of rabbitfish (Siganidae) from the Indo-West Pacific marine region

**DOI:** 10.1017/S0031182024001252

**Published:** 2024-10

**Authors:** Daniel C. Huston, Scott C. Cutmore, Thomas H. Cribb, Pierre Sasal, Russell Q.-Y. Yong

**Affiliations:** 1 Australian National Insect Collection, National Research Collections Australia, CSIRO, Canberra, ACT, Australia; 2 Queensland Museum, Biodiversity and Geosciences Program, South Brisbane, QLD, Australia; 3 School of the Environment, The University of Queensland, St Lucia, QLD, Australia; 4 CRIOBE, USR3278-EPHE/CNRS/UPVD/PSL, University of Perpignan Via Domitia, Perpignan, France; 5 Centre de Recherches Insulaires et Observatoire de l'Environnement (CRIOBE) BP1013, Papetoai, Moorea Polynésie Française; 6 Water Research Group, Unit for Environmental Sciences and Management, North-West University, Potchefstroom, South Africa

**Keywords:** *Atractotrema*, Atractotrematidae, Haploporata, Haploporoidea, *Siganus*, systematics, taxonomy

## Abstract

*Emprostiotrema* contains just 3 species: *E. fusum*, *E. kuntzi* and *E. sigani*. As adults, all 3 species infect rabbitfishes (Siganidae: *Siganus*). New collections from 11 species of *Siganus* from northern Australia, Indonesia, New Caledonia, French Polynesia, Palau and Japan enabled an exploration of species composition within this genus. Phylogenetic analyses demonstrate a deep distinction between 2 major clades; clade 1 comprises most of the sequences of specimens from Australia as well as all of those from Japan, Palau and New Caledonia and clade 2 comprises all sequences of specimens from French Polynesia, 2 sequences from Australia and the single sequence from Bali. In all analyses, both major clades have genetic structuring leading to distinct geographic lineages. Morphologically, specimens relating to clades 1 and 2 differ but overlap in body shape, oral sucker and egg size. Principle component analysis shows a general (but not complete) separation between specimens relating to the 2 clades. We interpret the 2 clades as representing 2 species: clade 1 is identified as *E. fusum* and is reported in this study from 10 species of siganids from Australia, Japan, Palau and New Caledonia; clade 2 is described as *E. gotozakiorum* n. sp., for all specimens from French Polynesia and rare specimens from Australia and Indonesia. We recognize *E. sigani* as a junior synonym of *E. fusum*. Although species of *Emprostiotrema* occur widely in the tropical Indo-Pacific, they have not been detected from Ningaloo Reef (Western Australia), the southern Great Barrier Reef or Moreton Bay (southern Queensland).

## Graphical abstract

**Figure:**
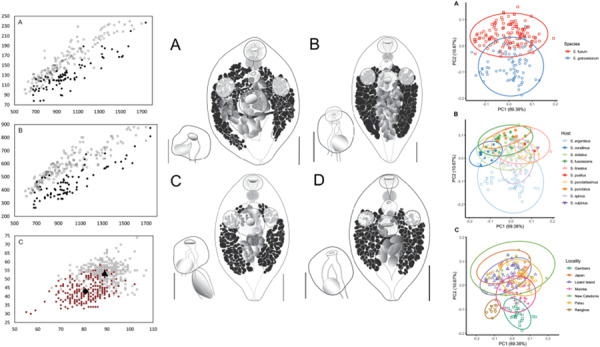

## Introduction

The digenean family Emprostiotrematidae Cianferoni and Ceccolini, 2021 is the smaller of 2 families making up the Haploporoidea Nicoll, 1914, comprising 4 genera and just 12 species. While species of the Haploporidae Nicoll, 1914 are cosmopolitan and occur in marine and freshwater ecosystems, emprostiotrematids have been found only in marine and estuarine systems of the Indo-West Pacific marine region (Overstreet and Curran, 2005; Bray *et al*., 2014; Andres *et al*., 2016; Huston *et al*., 2018*a*). Adult emprostiotrematids parasitize herbivorous or detritivorous fishes and are characterized morphologically by their possession of 2 caeca, 2 near-spherical testes that are symmetrically arranged, and vitelline follicles that form lobed aggregations in lateral fields (Overstreet and Curran, 2005; Andres *et al*., 2016; Huston *et al*., 2018*a*). This study focuses on the type-genus for the family, *Emprostiotrema* Cianferoni and Ceccolini, 2021, which was recently proposed as a replacement name for the junior homonym *Atractotrema* Goto and Ozaki, 1929 nec Cossmann, 1888 (see Ceccolini and Cianferoni, 2021). Ceccolini and Cianferoni (2021) proposed new combinations for the 3 recognized species of the genus – *Emprostiotrema fusum* (Goto and Ozaki, 1929) Cianferoni and Ceccolini, 2021; *Emprostiotrema kuntzi* (Ahmad, 1985) Cianferoni and Ceccolini, 2021; and *Emprostiotrema sigani* (Durio and Manter, 1969) Cianferoni and Ceccolini, 2021. Ceccolini and Cianferoni (2021) also proposed the replacement of the family group name Atractotrematidae with Emprostiotrematidae. All these modifications appear valid and are comprehensively adopted in this study.

All 3 species of *Emprostiotrema* have been reported from only rabbitfishes (Acanthuriformes: Siganidae: *Siganus*) and are distinguished from other emprostiotrematids by their possession of testes that are level with, or anterior to, the ventral sucker and an ovary that is nearly-to-distinctly post-testicular (Overstreet and Curran, 2005). The first species to be described was *E. fusum*, from *Siganus fuscescens* (Houttuyn), collected from off Takamatsu in the Seto Inland Sea of Japan (Goto and Ozaki, 1929). *Emprostiotrema sigani* was described from *Siganus lineatus* (Valenciennes), collected off Green Island on the central Great Barrier Reef, Australia and an unidentified *Siganus* sp. collected in New Caledonia (Durio and Manter, 1969). The most recently described species, *E. kuntzi*, was described from *Siganus argenteus* Quoy and Gaimard (as *Teuthis rostratus*), collected in the Arabian Sea, from off the west coast of India (Ahmad, 1985).

As part of a broad trematode sampling programme over the past 3 decades, a large number of emprostiotrematids were collected from siganids at locations across the Indo-west Pacific region. These new samples enable a re-examination of the *Emprostiotrema* species reported from the region, based on integrated morphological and molecular data. In this study, we propose 1 new species, synonymize 1 known species and demonstrate that *E. fusum* parasitizes a wide range of siganids and spans a broad geographic range in the western Pacific Ocean.

## Materials and methods

### Specimen collection and morphological analysis

**Figure 1.**
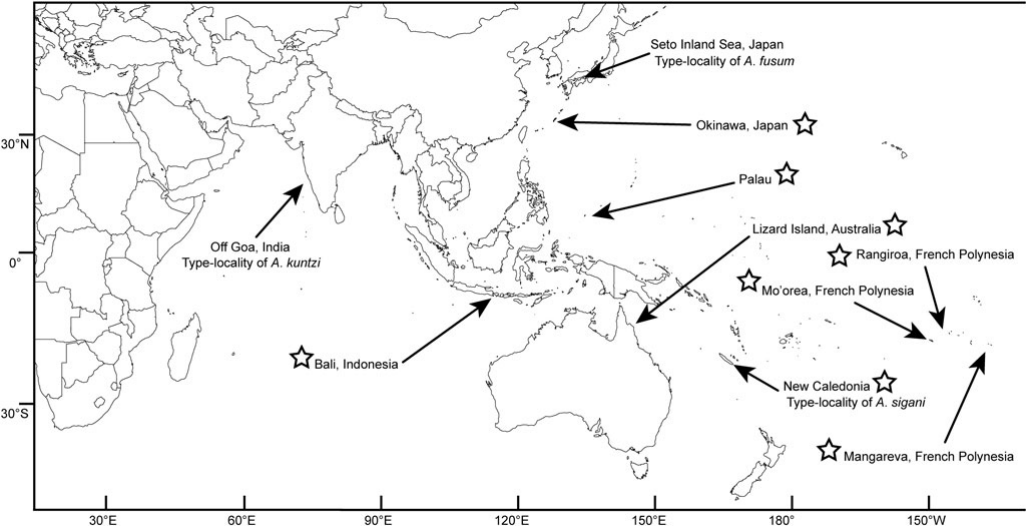
Map of known Indo-West Pacific collection localities for species of *Emprostiotrema*. Localities where specimens were obtained for the present study are indicated with a star symbol.

Siganid fishes were wild caught or (rarely) purchased from fish markets from 1986 to 2024 from locations across the Indo-West Pacific (Fig. 1). Fishes were euthanized by cranial pithing or overdose of Aqui-S™ anaesthetic and dissected as per the protocols of Cribb and Bray (2010). Trematodes were fixed without pressure in near-boiling saline and preserved in either 10% formalin or 70–80% ethanol. The validity of 1 host species, *Siganus canaliculatus* (Park), is controversial. The species, being sister to *S. fuscescens*, is extremely difficult to identify, with recent studies indicating minute (0.2%) genetic differences and instances of hybridization between the 2 (Zolkaply *et al*., 2021). Iwamoto *et al*. (2015) proposed synonymy of *S. canaliculatus* with *S. fuscescens*, though Zolkaply *et al*. (2021) indicate that more work needs to be done to resolve the relationship between the 2 taxa. Here we follow Eschmeyer's Catalog of Fishes (Fricke *et al*., 2024) in considering it a valid species. Host common names used in the text follow those used by FishBase.

Specimens prepared for morphological analyses were washed in fresh water, stained using Mayer's haematoxylin solution, de-stained using a dilute hydrochloric acid solution, neutralized using a dilute ammonium hydroxide solution, dehydrated using a graded ethanol series, cleared in methyl salicylate and mounted on slides in Canada balsam. Measurements were made using cellSens™ version 1.13 imaging software and an Olympus SC50 digital microscope camera mounted on an Olympus BX-53 compound microscope. Measurements, unless otherwise noted, are reported in micrometres (μm), and are given as a range with the mean in parentheses. Additional morphological data were obtained for a syntype of *E. fusum* (housed in the Meguro Parasitological Museum, Tokyo, Japan; accession number E0502) by analysing photomicrographs graciously provided by Professor Kazuo Ogawa. Measurement data for 37 features (see Supplementary Material) were imported into R (https://www.R-project.org), log-transformed and examined with principal component analyses (PCAs) of the covariance matrix and visualized using the package ggfortify (Tang and Horikoshi, 2016). Only those specimens that had a complete suite of measurements (i.e. included reliable measurements of all 37 selected features) were included in the PCA. (Thus, poor-quality specimens and hologenophores were excluded.) Drawings were made using a camera lucida, mounted on an Olympus BX-53 compound microscope. Drawings were digitized in Adobe Illustrator CS6. Type and voucher specimens are lodged in the Queensland Museum, Brisbane, Australia (QM), Museum National d'Histoire Naturelles, Paris, France (MNHN), Meguro Parasitological Museum, Tokyo, Japan (MPM), Museum Zoologicum Bogoriense, Bogor, Java, Indonesia (MZB); accession numbers are presented in the taxonomic section of this manuscript.

### Molecular sequencing and phylogenetic analysis

Molecular data were generated for 3 markers: the large subunit (28S), the second internal transcribed spacer (ITS2) of ribosomal RNA and the mitochondrial cytochrome c oxidase I (*cox*1) gene. Molecular specimens used were processed as hologenophores or whole paragenophores (sensu Pleijel *et al*., 2008). Genomic DNA were extracted using a modified phenol/chloroform technique (Sambrook and Russell, 2001). Primers, amplification and sequencing of the ITS2 and 28S regions follow Yong *et al*. (2016) and of the *cox1* region followed Wee *et al*. (2017). Dual direction Sanger sequencing was performed at the Australian Genome Research Facility, Brisbane. Contiguous sequences were assembled and edited with Geneious® version 11.0.5.

The *cox*1 and ITS2 datasets generated during this study were each aligned in MEGA 7 (Kumar *et al*., 2016), with UPGMA clustering for iterations 1 and 2; there are no previously published sequence data for either gene region for species of *Emprostiotrema* available on GenBank. The *cox*1 alignment was transferred to Mesquite v.3.31 (Maddison and Maddison, 2024), translated (echinoderm/flatworm mitochondrial code) and inspected for internal stop codons. All trimmed *cox*1 sequences were 474 base positions (bp). All codon positions in the *cox*1 dataset were evaluated for substitution saturation using the ‘Test of substitution saturation by Xia *et al*.’ function (Xia *et al*., 2003; Xia and Lemey, 2009) as implemented in DAMBE v. 7.2 (Xia, 2018); no significant substitution saturation was detected. Neighbour-joining analyses were conducted for each dataset using MEGA 7, with the following parameters: ‘Model/Method = No. of differences’, ‘Substitutions to Include = d: Transitions + Transversions’, ‘Rates among Sites = Gamma Distributed’ and ‘Gaps/Missing Data Treatment = Pairwise deletion’. Nodal support for the neighbour-joining analyses was estimated by performing 10 000 bootstrap replicates.

The partial 28S rDNA data generated during this study were aligned with emprostiotrematid sequence data available on GenBank (Table 1) using MUSCLE version 3.7 (Edgar, 2004) run on the CIPRES portal, with ClustalW sequence weighting and UPGMA clustering for iterations 1 and 2. The resultant alignment was refined by eye using Mesquite v.3.31; the ends of the alignment were trimmed, and indels constituting more than 3 bp and present in greater than 5% of the sequences in the dataset were removed. Bayesian inference analysis was performed using MrBayes version 3.2.7 (Ronquist *et al*., 2012) and maximum likelihood analysis using RAxML version 8.2.12 (Stamatakis, 2014), both run on the CIPRES portal. The best nucleotide substitution model was estimated using jModelTest version 2.1.10 (Darriba *et al*., 2012). Both the Akaike information criterion and Bayesian information criterion predicted the GTR + I + G as the best estimator; Bayesian inference and maximum likelihood analyses were conducted using the closest approximation to this model. Nodal support in the maximum likelihood analysis was estimated by performing 1000 bootstrap pseudoreplicates. Bayesian inference analysis was run over 10 000 000 generations (ngen = 10 000 000) with 2 runs each containing 4 simultaneous Markov Chain Monte Carlo (MCMC) chains (nchains = 4) and every 1000th tree saved. Bayesian inference analysis used the following parameters: ‘nst = 6’, ‘rates = invgamma’, ‘ngammacat = 4’, and the prior parameters of the combined dataset were set to ‘ratepr = variable’. Samples of substitution model parameters, and tree and branch lengths were summarized using the parameters ‘sump burnin = 3000’ and ‘sumt burnin = 3000’. Species of the Haploporidae were designated as functional outgroup taxa.

**Table 1.** Sequence data from GenBank included in this study

Species	Host	GenBank accession #	Reference
Haploporoidea			
Emprostiotrematidae			
*Emprostiotrema fusum*	*Siganus lineatus*	AY222267	Olson *et al*. (2003)
*Isorchis anomalus*	*Chanos chanos*	KU873018	Andres *et al*. (2016)
*Isorchis cannoni*	*Siganus lineatus*	MF803154	Huston *et al*. (2018*a*)
*Isorchis currani*	*Selenotoca multifasciata*	KU873016	Andres *et al*. (2016)
		MF803157	Huston *et al*. (2018*a*)
*Isorchis megas*	*Selenotoca multifasciata*	KU873015	Andres *et al*. (2016)
Haploporidae			
*Litosaccus brisbanensis*	*Mugil cephalus*	KM253765	Andres *et al*. (2014)
*Saccocoelium brayi*	*Chelon saliens*	FJ211234	Blasco-Costa *et al*. (2009)
*Saccocoelium tensum*	*Chelon auratus*	FJ211258	Blasco-Costa *et al*. (2009)

### Species recognition criteria

Species were distinguished using, as a starting point, the criteria for species delineation proposed by Bray *et al*. (2022).

## Results

### Collections

Specimens consistent with *Emprostiotrema* were collected from siganid fishes from 8 localities: Lizard Island on the northern Great Barrier Reef; off Bali in central Indonesia; off Nouméa, New Caledonia; from the main lagoon of Palau atoll; Okinawa Island in southern Japan; and from locations in 3 archipelagos in French Polynesia (Rangiroa, Tuamotu Archipelago; Moorea, Society Archipelago; Mangareva, Gambiers Archipelago). No emprostiotrematids were found in substantial samples of siganids from Heron Island on the southern Great Barrier Reef (*n* = 250), Moreton Bay in southeastern Queensland (*n* = 110) or Ningaloo Reef, Western Australia (*n* = 47). Table 2 summarizes the collections.

**Table 2.** Siganids examined at Indo-Pacific localities together with the prevalence of 2 species of *Emprostiotrema*

	Central Indo-Pacific								Eastern Indo-Pacific			
Host	BA	NI	HI	LI	MB	NC	OK	PA	GA	MO	MA	RA
*S. argenteus*	–	9 (0)	–	15 (2^a^/2^b^)	–	1 (0)	–	2 (0)	4 (3)	8 (1)	3 (0)	6 (2)
*S. canaliculatus*	1 (1)	–	–	–	–	–	–	–	–	–	–	–
*S. corallinus*	–	–	33 (0)	19 (10)	–	1 (0)	–	2 (0)	–	–	–	–
*S. doliatus*	–	–	26 (0)	24 (11)	–	6 (2)	–	3 (2)	–	–	–	–
*S. fuscescens*	–	15 (0)	45 (0)	4 (3)	110 (0)	3 (0)	6 (3)	–	–	–	–	–
*S. lineatus*	–	–	74 (0)	51 (25)	–	4 (4)	–	–	–	–	–	–
*S. puellus*	–	–	17 (0)	17 (0)	–	1 (0)	–	3 (1)	–	–	–	–
*S. punctatissimus*	–	–	–	12 (1)	–	–	–	2 (1)	–	–	–	–
*S. punctatus*	–	–	29 (0)	12 (1)	–	–	–	2 (1)	–	–	–	–
*S. spinus*	–	–	3 (0)	13 (0)	–	3 (0)	3 (0)	3 (1)	–	18 (5)	–	–
*S. trispilos*	–	11 (0)	–	–	–	–	–	–	–	–	–	–
*S. virgatus*	–	12 (0)	–	–	–	–	–	–	–	–	–	–
*S. vulpinus*	–	–	23 (0)	14 (1)	–	–	–	1 (0)	–	–	–	–

Number of fish followed by number of infected fish in parentheses. Legend: BA, Bali; NI, Ningaloo; HI, Heron Island; LI, Lizard Island; MB, Moreton Bay; NC, New Caledonia; OK, Okinawa; PA, Palau; GA, Gambiers; MA, Marquesas; MO, Moorea; RA, Rangiroa. The single Bali and all eastern Pacific records relate to *E. gotozakiorum* n. sp. All Central Pacific records relate to *E. fusum* except for in *S. argenteus* where ‘a’ denotes the prevalence of *E. fusum* and ‘b’ denotes *E. gotozakiorum* n. sp.

### Molecular results

**Figure 2.**
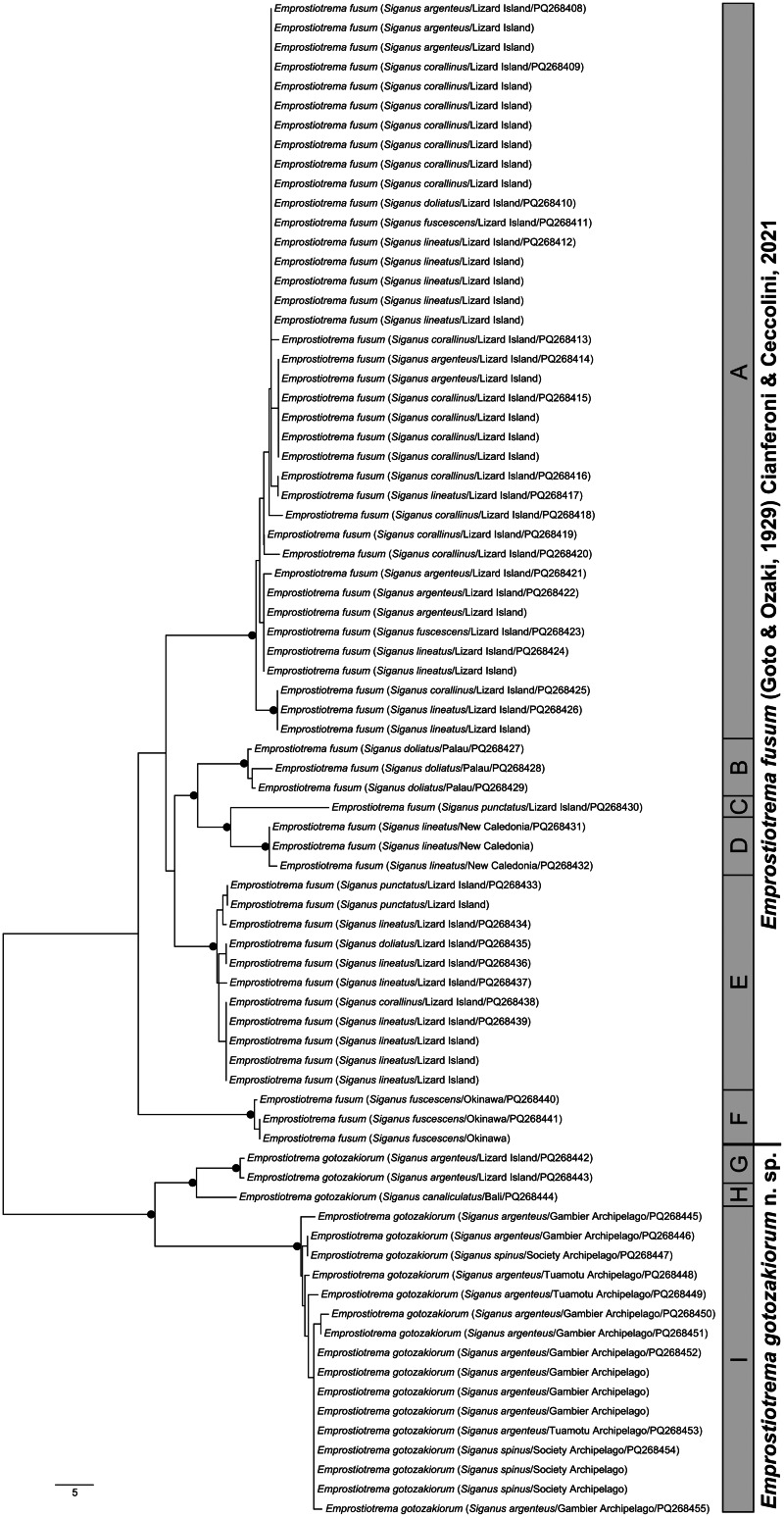
Phylogram from the unrooted neighbour-joining analysis of the cytochrome c oxidase subunit 1 (*cox*1) mtDNA dataset. Strongly supported nodes (>80) are indicated by a filled circle. The scale bar indicates the number of base differences.

A total of 78 *cox*1 sequences were generated for samples from all 8 localities, representing 13 host/locality combinations. Neighbour-joining analysis of these data (Fig. 2) resolved 9 lineages that differ by a minimum *P*-distance of 2.11% (10 bp), which we treat as 9 initial operational taxonomic units (OTUs A–I). All but 2 lineages were represented by multiple sequences, with OTU replication varying from 2 to 38 sequences. Inter-OTU *P*-distance differences ranged from 2.32 to 17.10% (11–81 bp) and intra-OTU variation ranged from 0 to 1.26% (0–6 bp). All 9 OTUs were restricted to single geographical localities with the exception of OTU I, which was found at sites in 3 French Polynesia archipelagos (Gambiers, Tuamotu and Society). The OTUs formed 2 major and deeply divided clades, clade 1 comprising 6 OTUs and clade 2 comprising 3 OTUs; these 2 clades differ by 13.10–17.10% (62–81 bp). Clade 1 corresponds to sequences of most specimens from Lizard Island (OTUs A, C and E), and all specimens from Palau (OTU B), New Caledonia (OTU D) and Japan (OTU F); inter-OTU *P*-distance differences within clade 1 range from 3.37 to 7.59% (16–36 bp). Clade 2 corresponds to all sequences of French Polynesian specimens (OTU I), 2 specimens from Lizard Island (OTU G) and the single sequenced specimen from Bali (OTU H); inter-OTU *P*-distance differences within clade 2 range from 2.32 to 7.38% (11–35 bp). Lizard Island was the only site from which multiple OTUs were found, with samples from this site resolving in 3 OTUs in clade 1 (OTUs A, C and E) and 1 OTU from clade 2 (OTU G). Notably, combinations of specimens relating to the 4 Lizard Island OTUs were found in total sympatry (same individual fish host).

**Figure 3.**
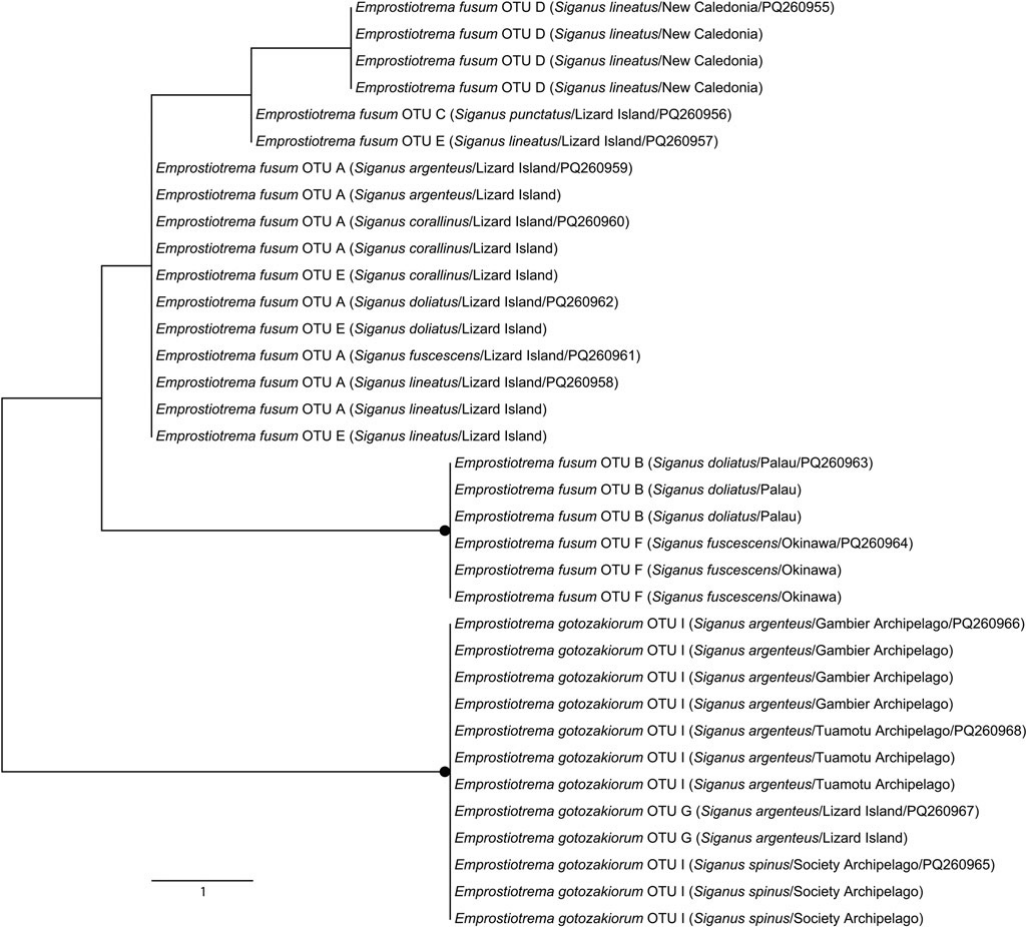
Phylogram from the unrooted neighbour-joining analysis of the internal transcribed spacer (ITS2) rDNA dataset. Strongly supported nodes (>80) are indicated by a filled circle. The scale bar indicates the number of base differences.

A total of 34 ITS2 sequences were generated, representing 8 of the 9 *cox*1 OTUs; no ITS2 data could be generated for OTU G (Bali). Neighbour-joining analysis of these data (Fig. 3) resolved the same 2 major clades as in the *cox*1 analysis with *P*-distance differences 1.40–2.10% (6–9 bp). These clades had less geographic structuring than was seen from the *cox*1 data. Sequences relating to clade 1 formed genotypes which differed by 0.23–1.40% (1–6 bp). Of 13 samples from Lizard Island, 11 were identical including multiple samples for each of OTUs A and E, which differed by 4.43–5.27% (21–25 bp) for the *cox*1 dataset. The remaining Lizard Island sequences were identical and differed from all others by 0.23% (1 bp). Four sequences from New Caledonia (OTU D) were identical and differed from the Lizard Island sequences by 0.23–0.47% (1–2 bp). Samples from Palau and Japan (OTUs B and F) were identical and differed from all other clade 1 sequences by 0.93–1.40% (4–6 bp). All sequences relating to clade 2, 10 sequences from French Polynesia (OTU I) and 2 sequences from Lizard Island (OTU G), were identical.

**Figure 4.**
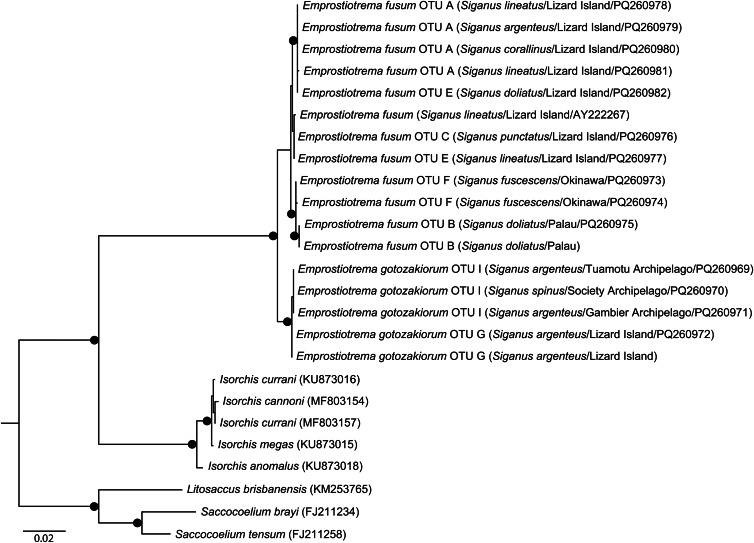
Relationships between species of the Emprostiotrematidae based on maximum likelihood phylogenetic analysis of the 28S dataset. Strongly supported nodes (Bayesian posterior probabilities >0.8 and maximum likelihood bootstrap values >80) are indicated by a filled circle. The scale-bar indicates expected number of substitutions per site.

28S data were generated from specimens from 7 of the 9 *cox*1 OTUs; no sequence data could be generated for OTU D (New Caledonia) or OTU H (Bali). Maximum likelihood and Bayesian inference analyses of the 28S dataset (Fig. 4) resolve *Emprostiotrema* as monophyletic, with sequences representing clades 1 and 2 forming well-supported clades that differ by 1.42–1.74% (18–22 bp). Sequences of OTUs A and E (both Lizard Island) were largely identical in the 28S dataset, with most differing inconsistently at a maximum of a *P*-distance of 0.08% (1 bp) and 2 sequences differing by 0.32–0.40% (4–5 bp). Sequences of OTUs B and F (Palau and Japan) formed a well-supported clade, differing from those from Lizard Island by 0.40–0.79% (5–10) bp. Within clade 2, there was no variation among sequences relating to OTU I (3 French Polynesian sites); the 2 sequences of OTU G (Lizard Island) differ from the French Polynesian data by 0.08% (1 bp).

### Morphological results

Specimens from Bali (OTU H) were excluded from morphological analysis as they had clearly been dead for some time at the point of fixation and had a distinctly distorted form. No hologenophore specimens were available for the OTUs C and G (both Lizard Island). Thus, morphological analyses were based on specimens representing 6 of the 9 OTUs (A, B, D, E, F and I), with a total of 222 specimens considered. Based on the overwhelmingly weighted molecular data (of the 52 Lizard Island specimens, just 2 specimens, not represented by hologenophores, belonged to clade 2), all Great Barrier Reef specimens were interpreted as relating to clade 1 (OTUs A, C or E).

**Figure 5.**
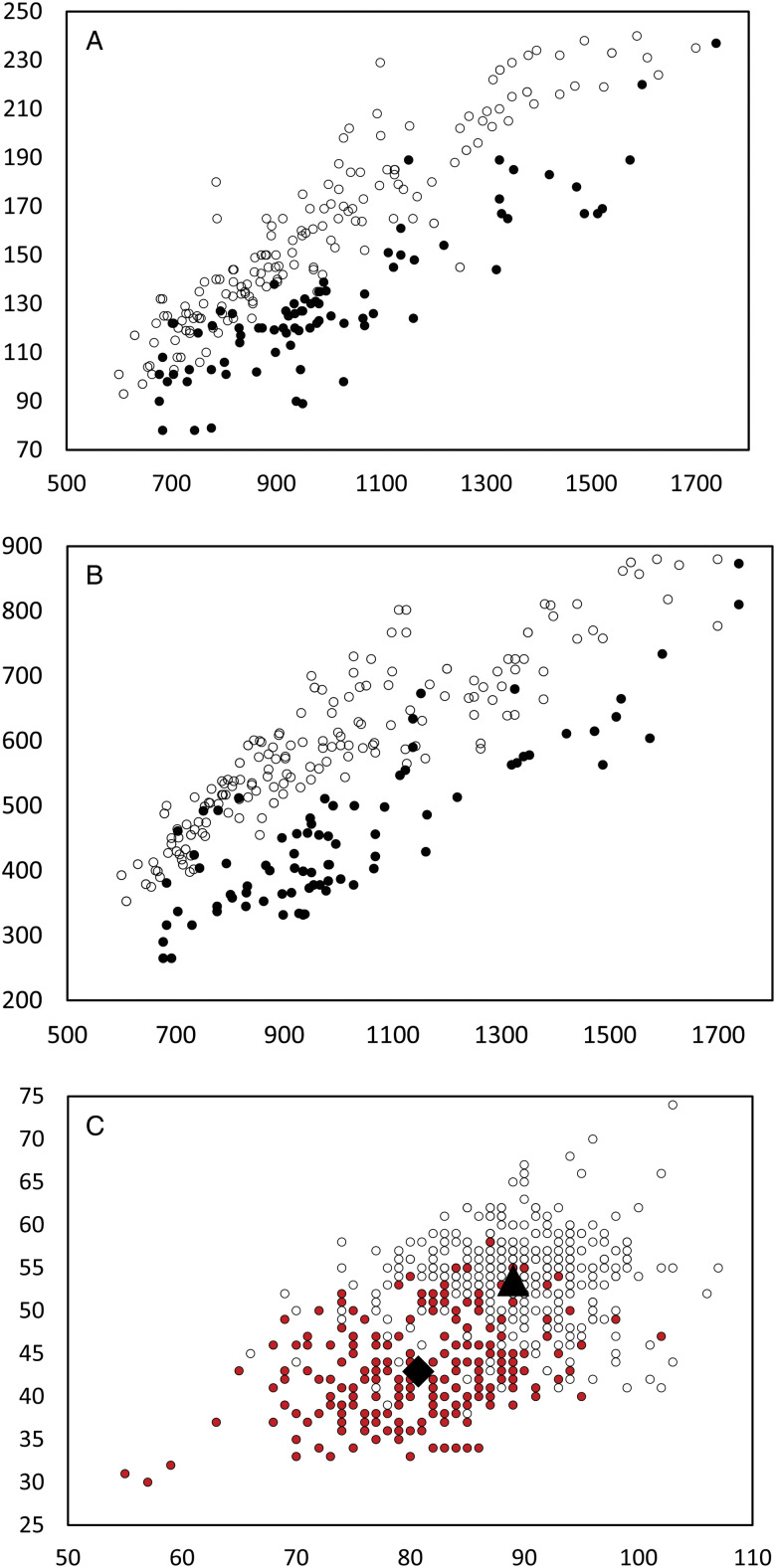
Morphometric comparison of *Emprostiotrema fusum* and *E*. *gotozakiorum* n. sp. (A) body length *vs* body width; (B) body length *vs* oral sucker width; (C) egg length *vs* width. Filled circle data points represent *E*. *fusum*; open circle data points represent *E*. *gotozakiorum* n. sp. The triangle in B represents mean egg size for *E*. *fusum*; the diamond represents mean egg size for *E*. *gotozakiorum* n. sp.

All specimens examined were broadly comparable, with no immediate distinctions suggesting the presence of multiple species. In the light of the molecular results, however, we considered a range of characters for samples from French Polynesia (OTU I) and all other sites (OTU A–F). Plotting of characters relative to body length and identified as relating to the 2 major clades suggest some morphological distinction; Fig. 5A shows body length *vs* width, Fig. 5B shows body length *vs* oral sucker width, and Fig. 5C shows egg size. In each there is a clear though incomplete separation between specimens relating to the 2 clades. Inspection of samples and plotting of measurements did not suggest any further morphological distinctions for subdivisions of the 2 clades.

### PCA analysis

**Figure 6.**
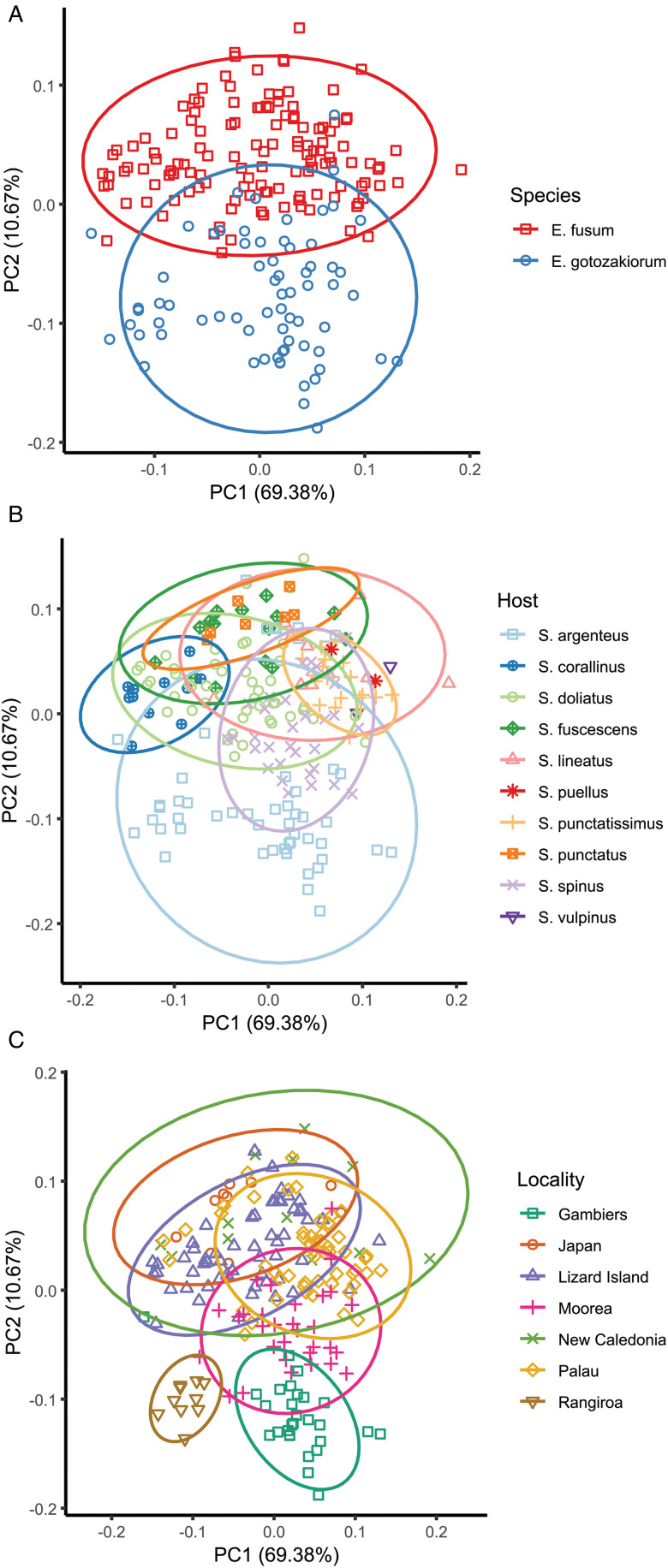
Principal component analysis (PCA) of morphometric data obtained from adult specimens of *Emprostiotrema* from multiple localities in the Indo-West Pacific. (A) Data points coded by species clade as informed by molecular data; (B) data points coded by host; (C) data points coded by collection locality. Ellipses represent 95% confidence levels.

PCA analysis of morphometric data was considered relative to the 2 major clades as identified by molecular analyses (Fig. 6A), host (Fig. 6B) and collection locality (Fig. 6C). The 95% confidence ellipses based on host suggested no informative break-up of the data. Ellipses based on locality suggested no distinction between localities in the western Pacific (Japan, Lizard Island, New Caledonia, Palau), some distinction between specimens from 2 French Polynesian localities (Rangiroa and Gambiers), and distinction between specimens from these 2 localities and all other more western Pacific localities. However, the Rangiroa and Gambiers ellipses overlapped with the Moorea ellipse, and in turn, the Moorea ellipse overlapped with all other western Pacific localities. The analysis based on just the 2 major clades (clade 1: Japan, Palau, Lizard Island and New Caledonia; clade 2: French Polynesia – 3 localities) produced a separation with overlap of ellipses between only 26% of the data points.

### Synthesis

Integrated consideration of host, molecular, morphological and morphometric analyses leads us to interpret the new specimens as representing 2 species. We conclude that host identity is uninformative in this system. Notably, *S. argenteus* is infected by specimens of both species at Lizard Island. PCA analysis also suggested no distinctions on the basis of host identity. Molecular data suggested a consistent division between clade 1 specimens (from Japan, Palau, New Caledonia and all but 2 from the Great Barrier Reef) relative to clade 2 specimens (from French Polynesia, Bali and 2 from Lizard Island). Sequences belonging to clade 1 have variable geographic division, with strong division in the *cox*1 dataset and limited division in the ITS2 and 28S datasets. Sequences belonging to clade 2 had strong geographic divisions in the *cox*1 dataset, and none or almost none in the ITS2 and 28S datasets, respectively. These levels of distinction within the 2 major clades, in the absence of correlation with morphological or host differences, are best interpreted as intra-specific variation. Morphology, in the form of 3 individual characters (body length *vs* body width; body length *vs* oral sucker width; egg length *vs* egg width), suggests distinction between specimens from Japan, Palau, New Caledonia and the Great Barrier Reef (consistent with clade 1) and those from the 3 French Polynesian sites (consistent with clade 2). PCA analysis suggested some division on the basis of geographical origin within French Polynesia. However, we infer that these distinctions cannot be interpreted as correlating with species distinctions, because separation within French Polynesian localities is not supported by any other data. Notably, *cox*1 sequences are now well-established to frequently show regional population differences in Indo-Pacific fish-infecting trematodes (Huston *et al*., 2021; Bray *et al*., 2022; Cutmore and Cribb, 2022; Cutmore *et al*., 2023; Pérez-Ponce de León *et al*., 2024), as seen here among clade 1 samples; the absence of differences between the 3 French Polynesian sites is thus intriguing. We infer that the PCA distinctions found between French Polynesian sites reflect either stochastic variation based on slight differences in handling, age of the parasites or geographical phenotypic plasticity.

Based on the integrated data, clade 1 specimens are identified as *E. fusum*. Notably, clade 1 includes new specimens from the type-host and relatively close to the type-locality of *E. fusum* [described by Goto and Ozaki (1929) from *S*. *fuscescens* off Takamatsu, Seto Inland Sea, Japan]; our new specimens from *S. fuscescens*, collected off Okinawa (approximately 1000 km to the south), conform closely to the original description of this species. Study of photographs of a syntype of *E. fusum* collected by Goto and Ozaki (MPM accession number E0502) further supports the conclusion that the new specimens from Okinawa are best interpreted as *E. fusum*. Durio and Manter (1969) described *E. sigani* based on specimens from *S*. *lineatus* caught off Green Island, central Great Barrier Reef, and from an unidentified *Siganus* sp. caught off Nouméa, New Caledonia. All new specimens of *Emprostiotrema* from *S. lineatus* from the Great Barrier Reef and from New Caledonia belong to clade 1. These new specimens correspond closely to the description of *E. sigani* of Durio and Manter (1969). Measurements provided by Durio and Manter (1969) to distinguish *E. sigani* completely overlap with those from *E. fusum* when our expanded dataset is considered, and we recognize *E. sigani* as a junior synonym of *E. fusum.*

Clade 2 is identified as a new species, which is recognizable on the basis of clear molecular distinctions and moderately clear morphological distinction. Notably, *E. fusum* and the new species were both collected from the same host species (and the same host individual) at Lizard Island, but there is as yet no evidence of overlap elsewhere in the range of the 2 species. We have not detected any morphological specimens consistent with the new species among the 58 mounted specimens from Lizard Island and conclude that it is an exceptionally rare species there.

#### Taxonomy

##### Family Emprostiotrematidae Cianferoni and Ceccolini, 2021 Genus *Emprostiotrema* Cianferoni and Ceccolini, 2021 Homonym: *Atractotrema* Goto and Ozaki, 1929 Syn *Atractotrema* Goto and Ozaki, 1929 nec Cossmann, 1888 *Emprostiotrema fusum* (Goto and Ozaki, 1929) Cianferoni and Ceccolini, 2021 (Fig. 7A–D)

**Figure 7.**
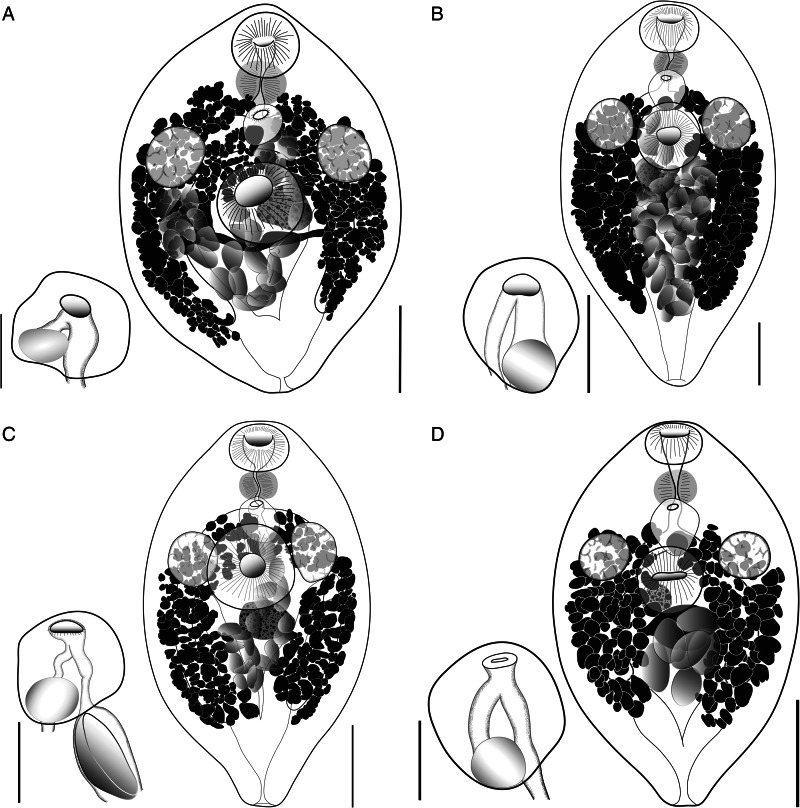
Plate showing whole-body ventrally mounted representatives of *Emprostiotrema fusum* and their terminal genitalia (hermaphroditic sacs), from (A) Japan ex *Siganus fuscescens* (accession no. MPM25300); (B) Lizard Island ex *S. doliatus* (QM G241330); (C) New Caledonia ex *S. doliatus* (MNH HEL 3003/MNH HEL 3001); and (D) Palau ex *S. punctatissimus* (QM G241418). Whole-mount scale-bars: A, B and D – 200 μm; C – 300 μm. Terminal genitalia scale-bars: A and B – 100 μm; C – 75 μm; D – 50 μm.

*Syn*: *Atractotrema fusum* Goto and Ozaki, 1929; *Atractotrema sigani* Durio and Manter, 1969; *E. sigani* (Durio and Manter, 1969) Cianferoni and Ceccolini, 2021.

*Type-host*: *Siganus fuscescens* (Houttuyn), dusky spinefoot (Acanthuriformes: Siganidae).

*Type-locality*: Takamatsu, Kagawa Prefecture, Seto Inland Sea, Japan.

*Reports*: Goto and Ozaki (1929); Yamaguti (1939); Durio and Manter (1969); Machida *et al*. (1970); Shen (1990); Cribb *et al*. (2001); Olson *et al*. (2003); Bakhoum *et al*. (2015); Motson *et al*. (2023).

#### New specimens

*New hosts*: *Siganus argenteus* (Quoy and Gaimard), streamlined spinefoot; *Siganus corallinus* Valenciennes, blue-spotted spinefoot; *Siganus puellus* (Schlegel), masked spinefoot; *Siganus punctatissimus* Fowler and Bean, peppered spinefoot; *Siganus punctatus* (Schneider and Forster), gold-spotted spinefoot; *Siganus spinus* (Linnaeus), little spinefoot; *Siganus vulpinus* (Schlegel and Müller), foxface (Acanthuriformes: Siganidae).

*Known hosts*: *Siganus doliatus* Guérin-Méneville, barred spinefoot; *S. fuscescens*, *S. lineatus* (Valenciennes), golden-lined spinefoot (Acanthuriformes: Siganidae).

*New localities*: off Nouméa, New Caledonia, Coral Sea (22°20′ S, 166°22′ E); off Lizard Island, northern Great Barrier Reef, Australia (14°40′ S, 145°27′ E); southern Palau lagoon, Palau (7°30′ N, 134°30′ E); Tomari fish market (26°13′ N, 127°40′ E), Naha, Okinawa Prefecture, Japan.

*Voucher material*: 136 voucher specimens (QM G 241301–440; MNHN 3001–4; MPM 25300).

*Prevalence*: See Table 2.

*Representative DNA sequences*: *cox1* mtDNA, 59 sequences (34 submitted to GenBank, PQ268408–41); ITS2 rDNA, 23 sequences (10 submitted to GenBank, PQ260955–64); partial 28S rDNA, 13 sequences (10 submitted to GenBank, PQ260973–82).

#### Description

[Based on 136 whole or partial, dorso-ventrally mounted specimens from Japan, Palau, Australia and New Caledonia. An extended set of measurements and indices covering 15 host/locality combinations are shown in Supplementary Table 1.] Body fusiform to lachrymiform, tapering anteriorly and posteriorly, 600–1699 × 353–880 (979 × 586); 1.4–2.2 (1.7) times longer than wide. Length of body post-point of maximal breadth 256–874 (476) or 38.6–60.5% (48.7%) of total body length. Tegumental spines <1 long, barely visible by light microscopy, arranged in transverse rows which wrap around entirety of body. Tegumental papillae or bristles not detected. Forebody 154–548 (293) long or 17.1–49.3% (30.1%) of total body length. Oral sucker prominent, ovoid, 74–210 × 93–255 (129 × 159), length/width ratio 0.6–1.3 (0.8). Mouth subterminal. Prepharynx not visible posterior to oral sucker (i.e. 0 length) in over half of specimens; when visible, length 3–44 (5); straight distance from mouth to pharynx 24–157 (69). Pharynx prominent, ovoid, 60–148 × 69–176 (91 × 105). Oesophagus variously conspicuous, sometimes barely discernible, straight or sinuous when visible, 0–103 (37) long, occupying 0–8.7% (3.9%) of total body length. Intestine bifurcates in anterior third of body, caeca run parallel to lateral contours of body, subequal in length in most specimens; left caecum 280–1074 (534); right caecum 273–1117 (542); longer caecum occupying 44.0–68.6% (56.1%) of body length. Ventral sucker prominent, ovoid to round, not pedunculate, 115–318 × 115–318 (197 × 193), length/width ratio 0.8–1.3 (1.0). Testes subspherical to spherical, entire, opposite, variously lateral to antero-lateral to ventral sucker, 72–237 × 66–197 (139 × 125), 158–533 (312) from anterior extremity, 331–1078 (580) from posterior extremity. Pre-testicular space 17.5–46.3% (31.3%) of total body length; post-testicular space 44.2–68.1% (58.0%) of total body length. Majority of male genital tract not observable until entering hermaphroditic sac. Hermaphroditic sac subspherical to diamond-shaped, containing seminal vesicle, generally medial, at level intermediate between pharynx and ventral sucker, 63–191 × 56–151 (116 × 102), 37–327 (197) or 6.1–31.7% (20.2%) of total body length from anterior extremity, 137–1178 (649) or 17.8–81.3% (65.4%) of total body length from posterior extremity. Seminal vesicle spherical, positioned in posterior region of hermaphroditic sac, 20–83 × 23–83 (43 × 48). Cirrus or other male intromittent organ absent. Male and female ducts confluent just posterior to common genital pore; no expansion or genital atrial formation apparent. Genital pore simple, medial, 47–357 (217) from lateral margins, 41–387 (206) or 6.7–31.0% (21.1%) of total body length from anterior extremity, 258–1354 (744) or 42.4–88.5% (75.6%) of total body length from posterior extremity. Ovary oblong, submedial, at or just posterior to level of testes and ventral sucker, 38–163 (91) × 37–183 (91 × 86). Oviduct and seminal receptacle not visible in any specimens. Oötype only visible if developing egg present, immediately posterior (and sometimes sinistral) to ovary. Uterus occupying intercaecal region of body, with eggs primarily concentrated posterior to testes, ovary and ventral sucker. Eggs oblong, 1–70 (17) in number, 63–107 × 38–74 (89 × 54), length/breadth ratio 1.3–2.5 (1.7). Vitelline follicles form variably sized clumps in 2 lateral fields, often anteriorly confluent, extending anteriorly to level of testes (just anteriorly exceeding testes in minority of specimens) and posteriorly to level of caecal termini, 110–413 (232) from anterior extremity, 40–366 (187) from posterior extremity, dorsoventrally overlapping and often obscuring caeca, testes and portions of uterus. Vitelline ducts arise from anterior portion of vitelline fields and unite medially, posterior to ovary. Excretory vesicle V-shaped, 319–1326 (663) long, arms pass parallel to lateral margins of body, closely overlapping caeca, terminating variously well anterior to immediately posterior margin of testes.

#### Remarks

The original description of *E. fusum* by Goto and Ozaki (1929) is somewhat lacking in detail, but the accompanying figure is of high quality and clearly shows the nature of the species. No holotype was designated by Goto and Ozaki (1929) in their original description; instead, 5 specimens were used for a syntype series (K. Ogawa, personal communication). Notably, the overall body size and dimensions of many of the features described by Goto and Ozaki (1929), and those of the syntype examined by us, are much larger than those seen in our specimens. This may be an artefact of flattening of specimens during fixation, a common practice in trematode taxonomy at the time Goto and Ozaki were active (see Huston *et al*., 2019). Beyond this general size difference, our specimens conform almost exactly in body plan and in all morphological features.

Durio and Manter (1969) differentiated *E. sigani* from *E. fusum* on the basis of a larger egg size, longer prepharynx and a body length half that of *E. fusum*. In our expanded concept of *E. fusum*, we find that the described dimensions of *E. sigani*, including body length and egg size, fall within the range of those of *E. fusum*. Indeed, Durio and Manter's description of egg size for *E. sigani* (80–103 × 50–62 μm) extensively overlapped those for *E. fusum* (82–88 × 50–58 μm), and merely indicated the presence of (and potential for) slightly larger eggs. When considered in light of the new collections from the type- and other locations, these differences disappear altogether. In addition, we consider prepharynx length a weak feature for species delineation, as it varies widely depending on how each specimen contracts when fixed. Most specimens we collected from the Great Barrier Reef, New Caledonia and Japan did not have a prepharynx visible posterior to the oral sucker. We therefore do not regard this, or any other feature used to differentiate *E. sigani* from *E. fusum*, as valid, and see no reason to consider them as separate species.

##### *Emprostiotrema gotozakiorum* n. sp. (Figs 8A–B and 9A–D)

**Figure 8.**
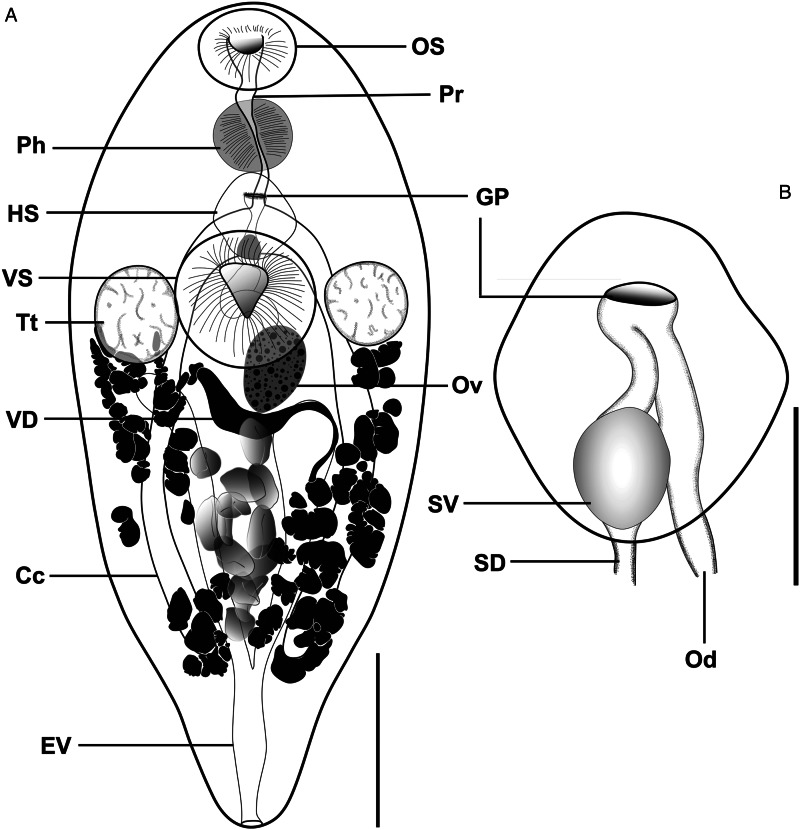
*Emprostiotrema gotozakiorum* n. sp. ex *Siganus argenteus*. (A) Whole-body ventral mount, holotype (accession no. MNH HEL3011). (B) Terminal genitalia (hermaphroditic sac), paratype (MNH HEL3009). Abbreviations: Cc, caeca; EV, excretory vesicle; GP, genital pore; HS, hermaphroditic sac; Od, oviduct; OS, oral sucker; Ov, ovary; Ph, pharynx; Pr, prepharynx; s.d., seminal duct; SV, seminal vesicle; Tt, testis; VD, vitelline duct; VS, ventral sucker. Scale-bars: A – 200 μm, B – 75 μm.

**Figure 9.**
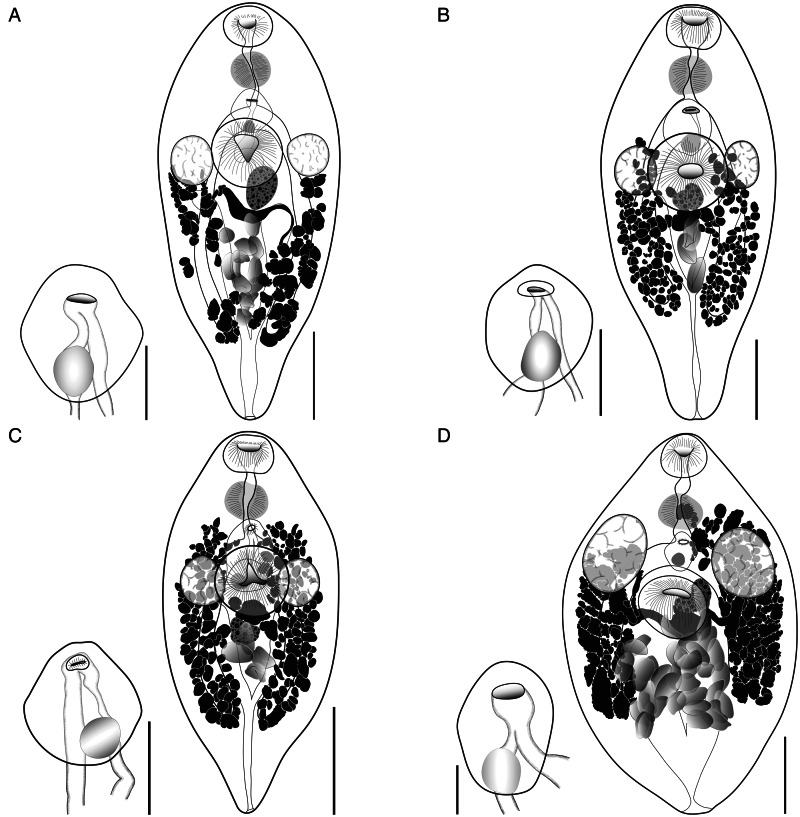
Plate showing whole-body ventrally mounted representatives of *Emprostiotrema gotozakiorum* n. sp. and their terminal genitalia (hermaphroditic sacs), from (A) Rangiroa, French Polynesia ex *Siganus argenteus* (MNH HEL3011/MNH HEL3009); (B) Moorea, French Polynesia ex *Siganus spinus* (MNH HEL3018/MNH HEL3026); (C) Mangareva, French Polynesia ex *S. argenteus* (MNH HEL3047); and (D) Bali, Indonesia ex *Siganus canaliculatus* (MZB Tr. 268). Whole-mount scale-bars: A, C, D – 200 μm, B – 250 μm. Terminal genitalia scale-bars: A, B, C – 75 μm, D – 100 μm.

*Type-host*: *Siganus argenteus* (Quoy and Gaimard), streamlined spinefoot (Acanthuriformes: Siganidae).

*Additional hosts*: *Siganus canaliculatus* (Park), white-spotted spinefoot; *S. spinus* (Linnaeus), little spinefoot (Acanthuriformes: Siganidae).

*Type-locality*: Rangiroa Atoll, Tuamotu Archipelago, French Polynesia (15°06′ S, 147°40′ W).

*Additional localities*: Moorea, Society Archipelago, French Polynesia (17°32′ S, 149°46′ W); Mangareva, Gambiers Archipelago, French Polynesia (23°07′ S, 134°58′ W); off Lizard Island, northern Great Barrier Reef, Australia (14°40′ S, 145°27′ E); off Kedonganan Beach, Jimbaran, southern Bali, Indonesia (8°46′ S, 115°08′ E).

*Type-material*: Holotype (MNHN HEL3005) and 12 paratypes (including 3 hologenophores) from Rangiroa, French Polynesia (MNHN 3006–64).

*Additional vouchers*: 69 vouchers from Gambiers and Moorea, French Polynesia (QM G241441–480); 3 vouchers from Bali, Indonesia (MZB Tr. 268–270).

*Prevalence*: See Table 2.

*Representative DNA sequences*: Representative DNA sequences: *cox1* mtDNA, 19 sequences (14 submitted to GenBank, PQ268442–55); ITS2 rDNA, 12 sequences (4 submitted to GenBank, PQ260965–68); partial 28S rDNA, 8 sequences (4 submitted to GenBank, PQ260969–72).

*ZooBank accession*: http://zoobank.org/NomenclaturalActs/6B856044-CFFB-41FD-ABA5-05E96A4DF247

*Etymology*: The species name is a compound of the names of 2 eminent Japanese parasitologists, Seitaro Goto and Yoshimasa Ozaki. This name honours their contributions to parasite taxonomy and their role as describers of the type-species of the Emprostiotrematidae, *Atractotrema* (now *Emprostiotrema*) *fusum*.

#### Description

[Based on 82 whole or partial, dorso-ventrally mounted specimens from Rangiroa, Gambiers and Moorea, French Polynesia (specimens from Bali, Indonesia were excluded from consideration). An extended set of measurements and indices covering 5 host/locality combinations are shown in Supplementary Table 1]. Body fusiform, tapering gently anteriorly and distinctly posteriorly, 677–1738 × 265–873 (1019 × 472), 1.5–2.8 (2.3) times longer than wide. Length of body post-point of maximal breadth 300–1060 (585) or 37.0–69.4% (57.3%) of total body length. Tegumental spines <1 long, barely visible by light microscopy, arranged in transverse rows which wrap around entirety of body. Tegumental papillae or bristles not detected. Forebody 210–594 (322) long or 24.8–41.7% (30.9%) of total body length. Oral sucker prominent, 65–202 × 78–237 (113 × 135), length/width ratio 0.6–1.3 (0.8). Mouth subterminal. Prepharynx not visible posterior to oral sucker (i.e. 0 length) in 7 of 82 (8.5%) specimens; when visible, length 2–67 (21); straight distance from mouth to pharynx 14–95 (66). Pharynx prominent, ovoid, 67–199 × 68–200 (104 × 108). Oesophagus variously conspicuous, straight or sinuous when visible, 19–93 (49) long, occupying 2.7–7.5% (4.7%) of total body length. Intestine bifurcates in anterior third of body, caeca run parallel to lateral contours of body, subequal in length in most specimens; left caecum 336–984 (581), right caecum 372–1036 (587); longer caecum occupying 45.9–70.3% (57.6%) of body length. Ventral sucker prominent, ovoid to round, not pedunculate, 124–352 × 117–366 (194 × 192), length/width ratio 0.8–1.3 (1.0). Testes subspherical to spherical, entire, paired, lateral, at level of ventral sucker, 65–262 × 47–207 (132 × 106), 236–740 (371) from anterior extremity, 262–1010 (584) from posterior extremity. Pre-testicular space 28.7–42.3% (34.6%) of total body length; post-testicular space 33.8–63.9% (55.2%) of total body length. Majority of male genital tract not observable until entering hermaphroditic sac. Hermaphroditic sac subspherical to diamond-shaped, containing seminal vesicle, generally medial, at level intermediate between pharynx and ventral sucker, 78–275 × 61–246 (137 × 117), 144–425 (241) or 17.2–30.6% (23.2%) of total body length from anterior extremity, 405–1189 (665) or 55.5–77.7% (64.6%) of total body length from posterior extremity. Seminal vesicle spherical, positioned in posterior region of hermaphroditic sac, 32–89 × 32–111 (54 × 52). Cirrus or other male intromittent organ absent. Male and female ducts confluent just posterior to common genital pore; no expansion or atrial formation apparent. Genital pore simple, medial, 165–459 (259) or 19.2–33.0% (24.9%) of total body length from anterior extremity, 460–1314 (769) or 64.7–83.3% (74.9%) of total body length from posterior extremity. Ovary oblong, submedial, at or just posterior to level of testes and ventral sucker, 49–245 × 38–152 (97 × 72). Oviduct and seminal receptacle not visible in any specimens. Oötype not visible in any specimens. Uterus occupying intercaecal region of body, with eggs primarily concentrated posterior to testes, ovary and ventral sucker. Eggs oblong, 1–77 (13) in number, 55–102 × 30–58 (81 × 43), length/breadth ratio 1.4–2.5 (1.9). Vitelline follicles form variably sized clumps in 2 lateral fields, occasionally anteriorly confluent, extending anteriorly to level of testes (just anteriorly exceeding testes in minority of specimens) and posteriorly to level of caecal termini, 137–666 (313) from anterior extremity, 93–412 (215) from posterior extremity, dorsoventrally overlapping and often obscuring caeca, testes and portions of uterus. Vitelline ducts arise from anterior portion of vitelline fields and unite medially, posterior to ovary. Excretory vesicle V-shaped, arms pass parallel to lateral margins of body, closely overlapping caeca, terminating immediately posterior to posterior margin of testes, 635–997 (822) long.

#### Remarks

*Emprostiotrema gotozakiorum* n. sp. is distinguished from its congeners by its body shape. Although all species of *Emprostiotrema* are generally spindle-to-teardrop-shaped, *A. gotozakiorum* typically has a slenderer body overall and a distinctly tapering hindbody, which leads to the highest mean length/maximum width ratio of any *Emprostiotrema* species [2.3 *vs* 1.7 for *E. fusum* (this study) and ~2.0 for *E. kuntzi*]. Correspondingly, *A*. *gotozakiorum* has a greater body length post-maximum breadth (both in absolute terms and as a proportion of total body length) and a greater distance of forebody features (ventral sucker, genital pore, hermaphroditic sac) to the posterior extremity than all other *Emprostiotrema* species.

## Discussion

### Species recognition

The incorporation of DNA sequence data in taxonomic studies of trematodes has enabled a nuanced interpretation of species delimitation over wide geographical ranges. Widespread but geographically structured species have now been characterized for multiple trematode families in the Indo-west Pacific (Huston *et al*., 2021; Bray *et al*., 2022; Cutmore and Cribb, 2022; Cutmore *et al*., 2023). However, in some cases, different markers have suggested conflicting interpretations, complicating species delimitation (e.g. Cutmore *et al*., 2021; Cribb *et al*., 2022; Wee *et al*., 2022). Overall, it is now clear that variability in the highly discriminant markers itself varies among families, genera and even species of single genera; there is seemingly no reliable yardstick that can presently be applied to delineate otherwise cryptic trematode species (e.g. Cutmore *et al*., 2021, 2023; Huston *et al*., 2021). In the current study, samples relating to *E. fusum* showed incongruence between the genes analysed. For the ribosomal regions, samples from Australia + New Caledonia and samples from Japan + Palau formed well-supported clades which differed by a *P*-distance of 0.93–1.40% (4–6 bp) in the ITS2 dataset and by 0.40–0.79% (5–10 bp) in the 28S dataset. Differences less than these are routinely found between morphologically distinct trematode species in the region (e.g. Trieu *et al*., 2015; Bray *et al*., 2018; Huston *et al*., 2018*b*; Huston *et al*., 2021); notably, Bray *et al*. (2023) used mitochondrial data to describe 2 species pairs (each distinct in morphology and host range) that had identical ribosomal data.

In contrast to ribosomal data, the *cox*1 data for specimens of *E. fusum* from the 4 Pacific localities form 6 well-supported clades (3 from Australia and 1 from each of New Caledonia, Japan and Palau), with geographical structuring different from that suggested by the ribosomal data; samples from Palau and New Caledonia formed a clade with 3 lineages of Lizard Island samples, sister to samples from Japan. Based on the *cox*1 data, the specimens of *E. fusum* could have been interpreted as 4 species (1 from Japan, 2 from Lizard Island and 1 from New Caledonia + Palau + Lizard Island). Only a handful of specimens of *E. gotozakiorum* were collected outside of French Polynesia, but, based on these samples, this new species demonstrates a topology that would have been predicted over such a range. In the *cox*1 analyses, samples from French Polynesia formed a well-supported clade, sister to a clade of specimens from Bali and Lizard Island; samples from French Polynesia and Lizard Island were almost identical in the ribosomal analyses. Although the level of intraspecific variation in the *cox*1 dataset, up to 7.38% (35 bp) and 7.59% (36 bp) within each of the 2 major clades, is high compared to a range of recent studies in the region (e.g. Cutmore and Cribb, 2022; Bray *et al*., 2023; Duong *et al*., 2023), it is lower than differences interpreted as intraspecific variation for several other species in the Indo-west Pacific. Magro *et al*. (2023) found variation of up to 8.9% (42 bp) for samples of *Paraschistorchis stenosoma* (Hanson, 1953) Blend, Karar & Dronen, 2017 in sympatry on the Great Barrier Reef; Cribb *et al*. (2024) found variation up to 11.2% (53 bp) for samples of *Prodistomum orientale* (Layman, 1930) Bray and Gibson, 1990 between Australia and Japan; and Cutmore *et al*. (2023) found variation of 12.6% (60 bp) for samples of *Transversotrema enceladi* Cutmore, Corner & Cribb, 2023 between Heron and Lizard Islands, both on the Great Barrier Reef. Overall, the final identification of all samples of clade 1 as representing *E. fusum* and all samples of clade 2 representing *E. gotozakiorum* was only possible by combining mitochondrial and ribosomal data interpretations, with a systematic consideration of the context of the data within each individual clade.

PCA analysis provided a general separation between the 2 species recognized here and 3 individual characters enabled a partial distinction between them. However, we consider reliable identification of these forms to be genotype-dependent; that is, molecular data provide a far more reliable basis for separation than do morphological characters. Should samples from new localities or hosts become available, a combined molecular and morphological assessment is desirable, but we predict that molecular data will provide the strongest evidence. In light of the results of this study, it may be possible to partly predict the identity of species in this genus by their geographic origin, but the presence of 2 species at Lizard Island where 1 is seemingly rare makes even this somewhat problematic.

### Mitochondrial populations

One striking feature of the *cox*1 dataset is the multiple lineages of *E. fusum* at Lizard Island; 3 lineages (OTUs A, C and E) were found at this location, with combinations of these lineages found in total sympatry (same individual fish); none of OTUs A, C and E are each other's sister clade. Analogous occurrences of multiple Lizard Island lineages have recently been reported recently, but few with such a complicated topology. Bray *et al*. (2022) found 2 lineages of *Preptetos zebravaranus* Bray, Cutmore & Cribb, 2022, Cutmore *et al*. (2023) found 2 lineages each for *Transversotrema fusilieri* Hunter and Cribb, 2012 and *T. iapeti* Cutmore, Corner & Cribb, 2023 and Magro *et al*. (2023) found 2 lineages for *P. stenosoma* (Hanson, 1953) Blend, Karar & Dronen, 2017; in all these cases, the 2 lineages were sister to each other. More comparable to the current topology is that of *Preptetos laguncula* Bray and Cribb, 1996. Bray *et al*. (2022) demonstrated that *P. laguncula* is represented by 2 clades at Lizard Island, 1 more closely related to samples from French Polynesia (Society and Gambiers Archipelagos) than to the other Lizard Island clade; as in the current study, the ribosomal data for these 2 clades were identical. Bray *et al*. (2022) speculated that the phenomenon may be a result of Lizard Island representing a ‘suture zone’, where previously isolated populations of *P. laguncula* had merged and the ribosomal DNA recombined while the mitochondrial populations remained distinct; the new data for *E. fusum*, as well as that reported for *P. stenosoma*, *P. zebravaranus*, *T. fusilieri* and *T. iapeti*, all support this interpretation.

Another potential key factor in play may be connectivity. It is well understood that host vagility is an important determinant in parasite dispersal ability; the more vagile a host, the higher the ability of the parasite to disperse widely across oceanic regions, while the retained connectivity between populations in turn limits genetic variability. If we take several of our sampling locations (Okinawa, Palau, Bali and the northern Great Barrier Reef) to represent the western boundary of the West Pacific region, French Polynesia represents the near-eastern extent of this region. The distances between these localities therefore represent biogeographical extremes in relation to 1 another, ones that are largely insurmountable for most inshore reef-dwelling species. *Siganus argenteus*, however, is the most vagile siganid species, being among the most pelagic of siganids and the only species with a long pelagic larval duration (Zolkaply *et al*., 2021). Such traits have enabled it to become, along with *S. spinus*, the most widespread siganid species in the Tropical Indo-West Pacific (TIWP).

The centre of radiation for much of TIWP biodiversity, including of the rabbitfish family Siganidae, is held to be the Coral Triangle, i.e. the region bound by Peninsular Malaysia, the Philippines and the Solomon Islands (Carpenter and Springer, 2005). It is therefore likely that species of *Emprostiotrema* also radiated in this region, dispersed eastwards along with the only hosts able to carry them and ultimately speciated in isolation in French Polynesia. In this interpretation, the presence of genotypes consistent with this Polynesian taxon in the western Pacific represents rare instances of ‘spillback’ of this Polynesian genotype, dispersing westwards in relatively small numbers in highly vagile hosts like *S. argenteus*. The westerly flowing trans-oceanic currents in the South Pacific split northwards and southwards upon encountering the Australian coastline in the vicinity of the southern Coral Sea. This current pattern may contribute to a biogeographical connectivity barrier, which may explain some of the observed absences and limited genetic variability between parasites from Polynesia and those from the northern and southern GBR.

It is known that disparities in ecology can lead to genetic separation due to both pre- and post-zygotic isolation mechanisms forming barriers to recombination; for instance, Williamson *et al*. (2024) demonstrated that some individuals of a widespread and ecologically versatile species being resident and others being highly migratory effectively form 2 populations which ultimately speciate, though they may remain morphologically cryptic and still often exist in sympatry. In the case of *Emprostiotrema* spp., genomic-scale analysis of the 2 *Emprostiotrema* species may reveal even deeper genetic divergences between them than is apparent from our ribosomal and *cox*1 mitochondrial data, facilitated by the greater dispersal capacity of *S. argenteus* relative to other *Siganus* species (Zolkaply *et al*., 2021). We speculate that the divergence of *E. gotozakiorum* closely tracks that of *S. argenteus* in the late Miocene (approximately 14.3 mya) and subsequent eastward dispersal to Polynesia. These divergences would effectively prevent *E. gotozakiorum* from recombining with *E. fusum* in the relatively rare occasions the former re-encounter the latter.

### Prevalence, distribution and host-specificity

Although we have examined 689 individual siganids, the distribution of these samples between 12 collection localities and 13 siganid species dilutes the capacity to draw robust inferences relating to prevalence. The sample sizes do seem sufficiently robust to allow the inference that species of *Emprostiotrema* do not occur on the southern Great Barrier Reef, at Moreton Bay or at Ningaloo Reef (Western Australia). The siganids examined at these 3 sites overlap with species found infected elsewhere, except for *S. trispilos* and *S. virgatus*, which have been examined only at Ningaloo Reef. Absence of *Emprostiotrema* species from sites where suitable hosts are abundant contrasts with the genuine widespread distributions demonstrated for other Indo-Pacific fish trematode species, e.g. *Elaphrobates chaetodontis* (Yamaguti, 1970) Yong, Cribb & Cutmore, 2021 reported from the Great Barrier Reef, Japan and French Polynesia (Cutmore and Cribb, 2022), *Gorgocephalus yaaji* Bray and Cribb, 2005 reported from eastern South Africa, Lizard Island and French Polynesia (Huston *et al*., 2021) and *Schikhobalotrema acutum* (Linton, 1910) Skrjabin and Guschanskaja, 1955 reported from the Gulf of Mexico to Australia (Pérez-Ponce de León *et al*., 2024). We speculate that the absences relate to the absence of suitable intermediate hosts; however, as discussed below, these remain completely unknown.

The overall prevalence of *E. fusum* in all susceptible siganids at sites at which it was found was 70/228 (30.7%) and is remarkably similar to the figure for *E. gotozakiorum* in French Polynesia (11/36, 30.5%). In contrast to these moderate prevalences, just 2 *cox*1 sequences were consistent with *E. gotozakiorum* compared to 50 sequences of *E. fusum* from Lizard Island, <4% of the specimens of *Emprostiotrema* at this locality. Both *E. gotozakiorum* sequences were from specimens of *S. argenteus*, one of the 2 known hosts from French Polynesia. It remains to be determined whether *E. gotozakiorum* has a narrower host range than *E. fusum* on the GBR; in French Polynesia it has been found in the only 2 siganids that occur there (*S. argenteus* and *S. spinus*). *Emprostiotrema gotozakiorum* is thus both nearly morphologically cryptic relative to *E. fusum* and much less abundant. Discovery of such rare species is clearly problematic as it is dependent on time-consuming and moderately expensive sequencing. Almost by definition, we have little understanding of the distribution and significance of rarity of marine fish parasites. However, as discussed above, such occurrences are likely at least partly explained by host population connectivity and dispersal ability.

The variation in the prevalence of *E. fusum* is interesting. Eight of 10 species of *Siganus* examined at Lizard Island (by far the best sampled site at which it is present) were infected by *E. fusum*; it was not found in robust samples of *S. puellus* (*n* = 17), or *S. spinus* (*n* = 13). However, both of those species were found infected in small fish samples at Palau (just 3 examined fishes of each species). Overall, every siganid species examined at sites where infections were present was infected in at least 1 locality.

The broad definitive host range of *E. fusum* (11 siganid species) is somewhat surprising in that ecological differences in host biology typically correlate with differences in digenean fauna (Miller *et al*., 2011). Although all species of *Siganus* form a monophyletic clade, these fishes are far from uniform in their behaviour and diet. Siganid species can be broadly divided into 2 general body types which have phylogenetic support: a ‘fusiform’ body [clades 1 and 2 *sensu* Kuriiwa *et al*. (2007), including *S. argenteus*, *S. canaliculatus*, *S*. *fuscescens* and *S*. *spinus*], associated with species that school on reef and algal flats, and a ‘deep’ body [clade 3 *sensu* Kuriiwa *et al*. (2007), including *S*. *corallinus*, *S*. *doliatus*, *S*. *lineatus*, *S*. *puellus*, *S*. *punctatissimus*, *S*. *punctatus* and *S*. *vulpinus*], associated with species that form pairs or small groups and feed near hard corals and within reef crevices (Kuriiwa *et al*., 2007; Fox and Bellwood, 2013; Hoey *et al*., 2013). Diets also differ among siganids, with some feeding primarily on algae, others feeding mostly on detrital aggregates, and *S*. *puellus* feeding primarily on sponges (Fox *et al*., 2009; Fox and Bellwood, 2013; Hoey *et al*., 2013). *Emprostiotrema fusum* parasitizes siganids of both body types, multiple algal and detrital feeding species, and even the sponge-feeding *S*. *puellus*. In contrast, *E. gotozakiorum* is known only from clades 1 and 2 species (*S*. *argenteus, S. canaliculatus* and *S*. *spinus*); notably, however *S. argenteus* and *S. spinus* are the only species of *Siganus* known to occur in French Polynesia (Siu *et al*., 2017), where *E. gotozakiorum* is most commonly found. *Emprostiotrema kuntzi* is only known from *S*. *argenteus*; this species, however, has not been reported since its original description (Ahmad, 1985) and the lack of additional reports from the Indian Ocean do not allow for commentary of the host-specificity of this species.

### Life cycles

The life cycles of *Emprostiotrema* species remain unknown. However, the ability of species of *Emprostiotrema* to parasitize siganids that occupy multiple ecological and dietary niches, coupled with a known intermediate host for another emprostiotrematid, *Isorchis cannoni* Huston, Cutmore & Cribb, 2017, allows inference of the cercarial dispersal and encystment strategy used by these digeneans. The cercariae of *I*. *cannoni* emerge from the intertidal gastropod *Clypeomorus batillariaeformis* Habe and Kosuge (Caenogastropoda: Cerithiidae) and swim for a short period before they encyst in the benthos where they are incidentally consumed by grazing *S*. *fuscescens* and *S*. *lineatus* (Cannon, 1978; Huston *et al*., 2018*a*). The variety of dietary niches occupied by hosts of *E. fusum* suggests that the cercariae of this species also encyst in the environment, likely with little site specificity. Although no intermediate hosts are yet known for any species of *Emprostiotrema*, cerithiids seem likely as indicated by *I*. *cannoni*, and Rissooidea or Truncatelloidea gastropods have also been suggested as potential hosts for emprostiotrematids, as these groups serve as intermediate hosts for haploporids (Andres *et al*., 2016). As with most digenean lineages, the next step in our understanding of emprostiotrematids is likely to be obtained through discovery of new intermediate host species.

## Supporting information

Huston et al. supplementary material 1 Huston et al. supplementary material

Huston et al. supplementary material 2 Huston et al. supplementary material

Huston et al. supplementary material 3 Huston et al. supplementary material

## Data Availability

Data underlying the present work, including R code and raw data used in principle component analysis, and an extended set of measurements of specimens summarized by host/locality combinations, have been uploaded as Supplementary Material.

## Supplementary material

The supplementary material for this article can be found at https://doi.org/10.1017/S0031182024001252.
