# Supplementary material for: Taxonomy and systematics of Emprostiotrema Cianferoni and Ceccolini, 2021 (Digenea: Emprostiotrematidae), parasites of rabbitfish (Siganidae) from the Indo-West Pacific marine region
Source: Parasitology. 2024 Nov 20;151(12):1336–50. doi: 10.1017/S0031182024001252 (PMC11894025; doi:10.1017/S0031182024001252)
Supplement: Huston et al. supplementary material 2 — Huston et al. supplementary material [file S0031182024001252sup002.docx]

**Supplemental Data for “Huston et al. Taxonomy and systematics of Emprostiotrema Cianferoni & Ceccolini, 2021 (Digenea: Emprostiotrematidae), parasites of rabbitfish (Siganidae) from the Indo-West Pacific marine region.”**

**R Code used for Principle Component Analyses.**

NOTE: the data in the supplemental files is presently formatted as an excel spreadsheet; it needs to be converted to a .csv file to use the code that follows.

##PCA R Code for Emprisiotrema MS##

##DC Huston, 4 June 2024##

##Prerequirements##

##data import##

##Set working Directory##

setwd("C:/Users/HUS033/OneDrive - CSIRO/Documents/Atractotrema/R")

data1=read.csv("Emprostiotrema Data used for PCA.csv")

names(data1)

##Log Transformations##

Locality=data1[,1]

Host=data1[,2]

Species=data1[,3]

hostno=data1[,4]

specno=data1[,5]

individual=data1[,6]

log.data1=log(data1[,7:43])

log.data1$Locality=data1[,1]

log.data1$Host=data1[,2]

log.data1$Species=data1[,3]

log.data1$hostno=data1[,4]

log.data1$specno=data1[,5]

log.data1$individual=data1[,6]

##Setting up PCAs##

##PCA1 = Raw morphometrics, all values##

PCA1=prcomp(~ bodylength + bodywidth + lengthbodypostmaxbreadth + oralsuckerlength +

oralsuckerbreadth + ventralsuckerlength + ventralsuckerbreadth +

forebodylength + hindbodylength + pharynxlength + pharynxbreadth +

leftcaecumlength + rightcaecumlength + leftestislength +

leftestisbreadth + righttestislength + righttestisbreadth +

distancelefttestistoanterior + distancerighttestisttoanterior +

pretesticularspace + distancelefttestistoposterior +

distancerighttestistoposterior + posttesticularspace + ovarylength +

ovarybreadth + hermsaclength + hermsacbreadth +

distancehermsactoanterior + distancehermsactoposterior +

distancehermsacleft + distancehermsacright + distancegenitalporeleft +

distancegenitalporeright + distancegenitalporeanterior +

distancegenitalporeposterior + distancevitanterior +

distancevitposterior, data=log.data1, center=TRUE, na.action=na.exclude)

summary(PCA1)

PCAloadings=PCA1$rotation

PCAscores=PCA1$x

screeplot(PCA1)

##visualise data##

library(ggfortify)

##PCA PLot for Localities ONly, no 95% ellipses##

SpeciesPlot2=autoplot(PCA1, data=log.data1, shape = "Locality", colour = "Locality", size=2)

print((SpeciesPlot2 + scale_shape_manual(values = c(0, 1, 2, 3, 4, 5, 6, 7, 8)))

+ theme_classic()) + scale_color_brewer(palette = "Dark2")

##PCA Plot which produces localities pluse 95% ellipses##

SpeciesPlotEllipse=autoplot(PCA1, data=log.data1, shape = "Locality", colour = "Locality", size=2) +

stat_ellipse() + aes(color=Locality)

print((SpeciesPlotEllipse + scale_shape_manual(values = c(0, 1, 2, 3, 4, 5, 6, 7, 8)))

+ theme_classic()) + scale_color_brewer(palette = "Dark2")

##PCA PLot for Host only, no 95% ellipses##

SPLocalPlot=autoplot(PCA1, data=log.data1, shape = "Host", colour = "Host", size=2)

print((SPLocalPlot + scale_shape_manual(values = c(0, 10, 1, 9, 2, 8, 3, 7, 4, 6, 5)))

+ theme_classic()) + scale_color_brewer(palette = "Paired")

##PCA plot for host with 95% ellipses##

SPLocalPlotEllipse=autoplot(PCA1, data=log.data1, shape = "Host", colour = "Host", size=2) +

stat_ellipse() + aes(color=Host)

print((SPLocalPlotEllipse + scale_shape_manual(values = c(0, 10, 1, 9, 2, 8, 3, 7, 4, 6, 5)))

+ theme_classic()) + scale_color_brewer(palette = "Paired")

##PCA Plot for Species ONly, No 95% ellipse##

SPSpeciesPlot=autoplot(PCA1, data=log.data1, shape = "Species", colour = "Species", size=2)

print((SPSpeciesPlot + scale_shape_manual(values = c(0, 1))) + theme_classic() + scale_color_brewer(palette = "Set1"))

##PCA code which produces the Species Plot with 95% Ellipses##

SPSpeciesPlotTestEllipse=(autoplot(PCA1, data=log.data1, shape = "Species", colour = "Species", size=2) +

stat_ellipse()) + aes(color=Species)

print((SPSpeciesPlotTestEllipse + scale_shape_manual(values = c(0, 1))) + theme_classic() + scale_color_brewer(palette = "Set1"))

##END##
