# Supplementary material for: Taxonomy and systematics of Emprostiotrema Cianferoni and Ceccolini, 2021 (Digenea: Emprostiotrematidae), parasites of rabbitfish (Siganidae) from the Indo-West Pacific marine region
Source: Parasitology. 2024 Nov 20;151(12):1336–50. doi: 10.1017/S0031182024001252 (PMC11894025; doi:10.1017/S0031182024001252)
Supplement: Huston et al. supplementary material 3 — Huston et al. supplementary material [file S0031182024001252sup003.docx]

Supplementary Table 2. Measurements of *Emprostiotrema fusum* and *Emprostiotrema gotozakiorum* summarised by host and locality combinations.

| **Species** | ***E. fusum*** | ***E. fusum*** | ***E. fusum*** | ***E. fusum*** | ***E. fusum*** | ***E. fusum*** | ***E. fusum*** | ***E. fusum*** | ***E. fusum*** | ***E. fusum*** | ***E. fusum*** | ***E. fusum*** | ***E. fusum*** | ***E. fusum*** | ***E. fusum*** | ***E. gotozakiorum*** | ***E. gotozakiorum*** | ***E. gotozakiorum*** | ***E. gotozakiorum*** | ***E. gotozakiorum*** |
| --- | --- | --- | --- | --- | --- | --- | --- | --- | --- | --- | --- | --- | --- | --- | --- | --- | --- | --- | --- | --- |
| **Locality** | **Okinawa** | **New Caledonia** | **New Caledonia** | **Lizard Is.** | **Lizard Is.** | **Lizard Is.** | **Lizard Is.** | **Lizard Is.** | **Lizard Is.** | **Lizard Is.** | **Palau** | **Palau** | **Palau** | **Palau** | **Palau** | **Moorea** | **Moorea** | **Gambiers** | **Rangiroa** | **Bali** |
| **Host** | ***S. fuscescens*** | ***S. doliatus*** | ***S. lineatus*** | ***S. doliatus*** | ***S. fuscescens*** | ***S. vulpinus*** | ***S. argenteus*** | ***S. lineatus*** | ***S. corallinus*** | ***S. doliatus*** | ***S. punctatissimus*** | ***S. doliatus*** | ***S. punctatus*** | ***S. spinus*** | ***S. puellus*** | ***S. spinus*** | ***S. argenteus*** | ***S. argenteus*** | ***S. argenteus*** | ***S. fuscescens*** |
| **n** | **10** | **9** | **6** | **4** | **6** | **2** | **7** | **6** | **11** | **22** | **21** | **14** | **7** | **9** | **2** | **27** | **8** | **35** | **13** | **3** |
| **Body length** | 679–1349 (1019) | 785–1440 (1042) | 609–792 (702) | 1262–1378 (1311) | 630–988 (885) | 600–718 (659) | 753–956 (871) | 817–1200 (905) | 1250–1699 (1469) | 881–1326 (1097) | 645–818 (735) | 713–1539 (1063) | 833–1125 (970) | 728–971 (841) | 655–735 (695) | 683–1137 (896) | 704–1325 (990) | 677–1738 (995) | 1319–1574 (1433) | 955–1040 (998) |
| **Body breadth** | 488–767 (668) | 517–811 (626) | 353–517 (430) | 588–707 (639) | 410–643 (572) | 393–433 (413) | 499–682 (580) | 481–711 (549) | 640–880 (770) | 504–726 (624) | 379–566 (463) | 409–875 (593) | 593–802 (679) | 455–600 (535) | 375–513 (444) | 316–634 (450) | 337–680 (500) | 265–873 (441) | 549–665 (593) | 570–641 (613) |
| **Maximal length/breadth** | 1.36–1.83 (1.52) | 1.46–1.99 (1.66) | 1.53–1.82 (1.64) | 1.83–2.12 (2.01) | 1.45–1.72 (1.55) | 1.53–1.66 (1.59) | 1.40–1.57 (1.51) | 1.57–1.79 (1.65) | 1.76–2.19 (1.91) | 1.53–1.90 (1.75) | 1.45–1.82 (1.60) | 1.59–2.02 (1.79) | 1.39–1.50 (1.43) | 1.43–1.73 (1.57) | 1.43–1.75 (1.59) | 1.53–2.31 (2.03) | 1.53–2.39 (2.01) | 1.97–2.82 (2.51) | 2.29–2.64 (2.40) | 1.62–1.68 (1.65) |
| **Body breadth as % body length** | 54.7–73.7 (66.4) | 50.2–68.5 (61.0) | 54.8–65.4 (61.1) | 47.2–54.7 (50.0) | 58.1–69.1 (64.9) | 60.3–65.5 (62.9) | 63.5–71.3 (66.5) | 56.0–63.8 (60.8) | 45.7–56.7 (52.5) | 52.6–65.2 (57.2) | 54.8–69.2 (62.9) | 49.4–62.7 (56.0) | 66.6–72.2 (70.0) | 57.7–70.2 (63.8) | 57.3–69.8 (63.5) | 43.3–65.5 (49.7) | 41.8–65.5 (51.3) | 35.5–50.7 (40.1) | 37.9–43.7 (41.8) | 59.7–61.6 (60.7) |
| **Body length post-max breadth** | 301–675 (483) | 367–685 (503) | 310–403 (364) | 603–704 (638) | 293–517 (426) | 307–364 (336) | 370–453 (401) | 391–554 (478) | 580–874 (728) | 398–607 (508) | 292–470 (374) | 327–754 (512) | 398–534 (468) | 337–507 (418) | 256–370 (313) | 300–582 (475) | 367–837 (542) | 405–935 (592) | 776–1060 (888) | 416–500 (458) |
| **% body length post-max breadth** | 39.4–58.5 (47.1) | 44.0–51.9 (48.4) | 48.0–60.1 (51.9) | 46.6–51.1 (48.6) | 44.3–52.3 (48.0) | 50.7–51.2 (50.9) | 42.4–49.1 (46.1) | 46.2–60.5 (53.2) | 45.2–54.1 (49.5) | 38.6–56.4 (46.5) | 45.3–59.0 (50.8) | 43.2–54.7 (48.1) | 46.5–50.4 (48.2) | 45.9–55.6 (49.6) | 39.1–50.3 (44.7) | 43.9–59.8 (53.0) | 39.7–68.7 (55.2) | 37.0–69.4 (60.2) | 57.9–67.3 (61.9) | 43.6–48.1 (45.8) |
| **Oral sucker length** | 98–208 (170) | 87–210 (155) | 76–110 (90) | 147–166 (156) | 84–133 (111) | 83–88 (86) | 103–121 (111) | 102–151 (122) | 145–194 (180) | 119–206 (151) | 74–107 (93) | 91–191 (127) | 103–151 (128) | 86–108 (96) | 82–98 (90) | 78–145 (106) | 77–182 (113) | 65–202 (113) | 114–156 (131) | 95–128 (108) |
| **Oral sucker breadth** | 132–229 (188) | 140–232 (184) | 93–140 (122) | 193–217 (203) | 117–162 (145) | 101–108 (105) | 124–159 (142) | 133–163 (145.5) | 145–255 (219) | 150–226 (179) | 97–139 (118) | 120–233 (164) | 137–185 (161) | 119–145 (134) | 104–119 (112) | 78–161 (119) | 101–189 (139) | 90–237 (135) | 144–189 (171) | 110–145 (125) |
| **Oral sucker breadth/length** | 0.98–1.35 (1.12) | 1.03–1.61 (1.22) | 1.17–1.54 (1.36) | 1.26–1.33 (1.30) | 1.22–1.39 (1.31) | 1.22–1.23 (1.22) | 1.20–1.34 (1.28) | 1.08–1.31 (1.20) | 0.76–1.40 (1.23) | 0.87–1.42 (1.19) | 1.14–1.45 (1.28) | 1.20–1.40 (1.30) | 1.21–1.34 (1.26) | 1.31–1.51 (1.41) | 1.21–1.27 (1.24) | 0.75–1.35 (1.14) | 0.75–1.43 (1.00) | 0.76–1.67 (1.22) | 0.67–0.87 (0.77) | 1.13–1.18 (1.16) |
| **Oral sucker length as % body length** | 13.87–19.55 (16.63) | 9.65–20.13 (15.04) | 11.25–15.15 (12.87) | 11.81–12.61 (12.15) | 10.43–14.93 (12.67) | 12.26–13.83 (13.04) | 11.53–14.08 (12.83) | 12.24–14.39 (13.58) | 10.88–15.20 (12.29) | 12.12–16.62 (13.75) | 10.50–15.31 (12.62) | 10.86–12.97 (11.98) | 12.20–13.62 (13.19) | 10.06–12.22 (11.40) | 12.52–13.33 (12.93) | 9.22–17.31 (11.86) | 9.60–13.74 (11.37) | 8.26–13.11 (10.55) | 7.82–9.92 (9.02) | 9.81–9.95 (9.88) |
| **Ventral sucker length** | 140–258 (210) | 164–307 (228) | 133–164 (151) | 219–237 (230) | 132–205 (184) | 124–141 (133) | 159–202 (178) | 173–228 (191) | 197–318 (270) | 179–310 (226) | 115–174 (145) | 143–283 (205) | 188–229 (208) | 142–175 (161) | 131–162 (147) | 124–239 (168) | 136–275 (194) | 132–352 (199) | 221–273 (235) | 153–170 (160) |
| **Ventral sucker breadth** | 138–269 (219) | 160–310 (220) | 132–147 (138) | 227–252 (236) | 125–199 (177) | 125–139 (132) | 162–197 (176) | 148–180 (161) | 234–304 (267) | 185–302 (233) | 114–161 (140) | 142–283 (201) | 162–211 (197) | 133–162 (150) | 132–155 (144) | 133–246 (174) | 126–303 (197) | 117–366 (187) | 222–277 (244) | 171–225 (194) |
| **Ventral sucker breadth/length** | 0.94–1.24 (1.05) | 0.91–1.06 (0.97) | 0.84–1.02 (0.92) | 0.97–1.11 (1.03) | 0.90–1.03 (0.96) | 0.99–1.01 (1.00) | 0.93–1.08 (0.99) | 0.79–0.97 (0.84) | 0.87–1.19 (1.00) | 0.93–1.11 (1.03) | 0.87–1.07 (0.97) | 0.92–1.06 (0.98) | 0.83–1.03 (0.95) | 0.88–0.98 (0.93) | 0.96–1.01 (0.98) | 0.91–1.18 (1.04) | 0.97–1.23 (1.05) | 0.75–1.10 (0.93) | 0.87–1.13 (0.98) | 1.12–1.32 (1.21) |
| **Ventral sucker length as % body length** | 16.8–23.5 (20.7) | 18.2–27.9 (22.2) | 20.6–22.6 (21.5) | 16.5–18.8 (17.8) | 19.5–21.8 (20.8) | 19.6–20.7 (20.2) | 19.5–21.5 (20.5) | 19.0–23.1 (21.3) | 14.9–19.8 (18.4) | 17.8–23.4 (20.7) | 17.4–22.8 (19.7) | 17.4–22.5 (19.4) | 19.8–23.2 (21.6) | 18.0–21.3 (19.2) | 20.0–22.0 (21.0) | 15.7–23.0 (18.6) | 16.9–23.4 (19.7) | 16.8–20.5 (18.7) | 15.3–17.3 (16.5) | 15.1–16.0 (15.6) |
| **Ventral sucker area** | 60.7–211.2 (149.1) | 82.4–298.8 (161.9) | 56.8–71.1 (65.5) | 160.2–180.4 (169.8) | 51.8–121.2 (104.2) | 48.7–61.5 (55.1) | 83.9–118.6 (98.8) | 83.2–128.9 (96.7) | 145.4–290.2 (228.1) | 104.0–283.3 (168.3) | 41.2–87.0 (64.2) | 63.8–251.5 (135.3) | 99.7–151.0 (129.2) | 59.3–89.0 (76.4) | 54.3–78.8 (66.6) | 53.3–184.6 (94.1) | 53.8–238.3 (128.4) | 48.5–391.9 (128.9) | 161.0–226.3 (180.9) | 82.2–120.1 (98.0) |
| **Length VS / Length OS** | 1.12–1.51 (1.25) | 1.25–2.26 (1.52) | 1.49–1.90 (1.69) | 1.37–1.59 (1.47) | 1.46–1.95 (1.67) | 1.49–1.60 (1.55) | 1.50–1.69 (1.60) | 1.42–1.75 (1.57) | 1.06–1.72 (1.51) | 1.30–1.71 (1.51) | 1.30–1.98 (1.57) | 1.47–1.92 (1.62) | 1.46–1.90 (1.64) | 1.53–1.94 (1.69) | 1.60–1.65 (1.63) | 1.28–2.07 (1.67) | 1.52–2.30 (1.75) | 1.38–2.40 (1.79) | 1.49–2.05 (1.81) | 1.33–1.61 (1.49) |
| **Breadth VS / Breadth OS** | 1.05–1.25 (1.16) | 0.97–1.36 (1.20) | 0.99–1.46 (1.15) | 1.11–1.19 (1.16) | 1.07–1.42 (1.22) | 1.24–1.29 (1.26) | 1.13–1.35 (1.24) | 1.01–1.26 (1.11) | 1.12–1.65 (1.23) | 1.13–1.57 (1.30) | 0.98–1.42 (1.19) | 1.16–1.29 (1.22) | 1.14–1.36 (1.23) | 1.03–1.19 (1.12) | 1.27–1.30 (1.29) | 1.17–1.83 (1.47) | 1.25–1.60 (1.39) | 1.06–1.79 (1.36) | 1.28–1.69 (1.45) | 1.55–1.55 (1.55) |
| **Forebody length** | 272–548 (415.4) | 154–445 (309) | 170–231 (199) | 313–381 (343) | 200–285 (263) | 189–220 (204.5) | 241–274 (262) | 233–334 (256) | 316–448 (398) | 249–411 (305.5) | 189–259 (233) | 202–427 (295) | 224–377 (295) | 236–292 (257) | 207–222 (214.5) | 225–403 (295) | 232–532 (333) | 210–594 (321) | 333–419 (384) | 310–381 (352) |
| **Forebody as % body length** | 32.9–49.3 (40.9) | 17.1–39.9 (29.8) | 24.6–31.8 (28.4) | 24.8–27.6 (26.5) | 26.6–32.0 (29.9) | 30.6–31.5 (31.1) | 28.3–32.0 (30.2) | 26.8–30.5 (28.3) | 24.5–31.8 (27.1) | 24.2–32.4 (27.9) | 27.9–36.6 (31.8) | 21.6–33.4 (28.0) | 26.5–33.9 (30.3) | 28.7–33.4 (30.6) | 30.2–31.6 (30.9) | 28.2–41.7 (32.9) | 29.9–40.2 (33.8) | 26.6–33.0 (29.8) | 24.8–28.3 (26.9) | 32.5–36.6 (34.5) |
| **Hindbody length** | 216–555 (393.3) | 274–782 (528) | 283–415 (356) | 741–791 (758) | 298–492 (424.5) | 283–345 (314) | 329–466 (415) | 388–623 (448) | 664–953 (787) | 431–679 (557) | 293–401 (345) | 352–823 (556) | 384–541 (452) | 344–516 (419) | 303–328 (315.5) | 270–611 (453) | 256–663 (469) | 346–908 (513) | 769–932 (845) | 477–494 (486) |
| **Hindbody as % body length** | 27.5–49.7 (38.3) | 34.9–62.0 (50.0) | 46.3–53.9 (50.7) | 57.4–58.7 (57.8) | 44.9–52.8 (47.9) | 47.2–48.1 (47.6) | 43.7–49.7 (47.5) | 47.5–51.9 (49.3) | 48.9–56.1 (53.5) | 43.7–56.6 (50.8) | 42.1–51.6 (47.0) | 46.7–59.2 (52.0) | 44.2–49.7 (46.6) | 45.2–53.1 (49.8) | 44.6–46.3 (45.4) | 36.8–56.3 (50.0) | 36.4–56.3 (47.2) | 46.9–54.8 (51.4) | 57.5–61.3 (59.1) | 47.5–49.9 (48.7) |
| **Oesophagus straight length** | 11–49 (33.5) | 22–78 (42) | 16–51 (24) | 35–44 (39.5) | 0–57 (39) | 19–41 (30) | 21–41 (33) | 19–43 (33) | 28–66 (47) | 11–46 (32) | 12–51 (34) | 22–103 (47) | 17–47 (35) | 24–53 (40) | 31–44 (37.5) | 28–66 (48) | 24–78 (44.9) | 19–93 (44) | 52–82 (64.5) | 16–22 (19) |
| **Oesophagus straight length as % body length** | 1.61–7.22 (3.41) | 2.03–8.65 (4.23) | 2.20–6.44 (3.37) | 2.94–3.25 (3.13) | 0.00–6.40 (4.17) | 3.17–5.71 (4.44) | 2.79–4.66 (3.76) | 2.24–4.36 (3.61) | 1.76–4.25 (3.18) | 1.06–4.30 (2.89) | 1.73–7.23 (4.63) | 2.40–6.76 (4.29) | 2.01–5.36 (3.60) | 3.30–5.81 (4.76) | 4.73–5.99 (5.36) | 3.68–7.46 (5.41) | 3.09–5.89 (4.41) | 2.75–6.32 (4.13) | 3.42–5.57 (4.56) | 2.09–2.12 (2.10) |
| **Prepharynx (distance OS to pharynx)** | 0–4 (0.7) | 0–0 (0) | 0–0 (0) | 0–11 (2.75) | 0–10 (3) | 6–9 (7.5) | 0–7 (1) | 0–0 (0) | 0–42 (5) | 0–28 (6) | 0–16 (8) | 0–44 (13) | 0–0 (0) | 7–21 (15) | 0–15 (7.5) | 0–30 (16) | 12–27 (21) | 0–67 (25) | 0–30 (16) | 32–46 (41) |
| **Straight distance mouth to pharynx** | 26–107 (69) | 34–84 (52) | 32–68 (51) | 83–102 (90.75) | 58–68 (64) | 45–56 (50.5) | 57–70 (63) | 35–77 (55) | 52–157 (92) | 55–114 (79) | 44–73 (59) | 52–122 (84) | 24–78 (57) | 59–82 (74) | 49–65 (57) | 2–90 (57) | 21–80 (57.3) | 38–94 (68) | 45–95 (78.8) | 91–102 (98) |
| **Pharynx length** | 84–148 (123) | 90–131 (114) | 68–98 (81) | 96–113 (105) | 60–102 (91) | 64–69 (67) | 84–104 (91) | 81–104 (88) | 94–127 (116) | 64–114 (86) | 60–87 (74) | 63–116 (82) | 84–118 (98) | 70–86 (77) | 65–74 (70) | 77–131 (97) | 67–154 (108) | 72–199 (102) | 109–136 (124) | 94–109 (99) |
| **Pharynx breadth** | 85–176 (137) | 98–141 (123) | 83–105 (95) | 113–123 (117) | 73–112 (102) | 69–79 (74) | 96–108 (102) | 88–112 (100) | 111–147 (135) | 87–126 (101) | 72–99 (89) | 76–128 (101) | 94–123 (107) | 87–101 (93) | 72–86 (79) | 75–121 (101) | 73–125 (100) | 68–200 (107) | 114–139 (130) | 111–132 (120) |
| **Pharynx length as % body length** | 9.6–13.5 (12.2) | 8.7–14.1 (11.2) | 9.8–14.2 (11.5) | 7.8–8.7 (8.2) | 9.5–11.1 (10.3) | 9.6–10.7 (10.1) | 9.9–11.2 (10.4) | 8.7–10.7 (9.8) | 6.8–9.2 (7.9) | 6.8–10.2 (7.9) | 8.6–12.7 (10.0) | 6.8–9.1 (7.8) | 9.4–10.6 (10.1) | 8.2–10.8 (9.2) | 9.9–10.1 (10.0) | 8.1–15.3 (10.9) | 8.3–13.9 (10.9) | 8.1–12.5 (9.5) | 7.2–9.5 (8.6) | 11.3–11.6 (11.5) |
| **Pharynx breadth / length** | 0.96–1.28 (1.12) | 0.97–1.19 (1.08) | 1.06–1.35 (1.18) | 1.04–1.20 (1.12) | 1.05–1.22 (1.13) | 1.08–1.14 (1.11) | 1.04–1.18 (1.13) | 1.08–1.20 (1.13) | 1.07–1.32 (1.17) | 0.99–1.42 (1.19) | 1.07–1.35 (1.22) | 1.10–1.39 (1.24) | 0.98–1.14 (1.10) | 1.12–1.33 (1.22) | 1.11–1.16 (1.13) | 0.81–1.23 (1.05) | 0.79–1.12 (0.95) | 0.86–1.23 (1.06) | 0.88–1.24 (1.05) | 1.18–1.26 (1.22) |
| **Pharynx breadth / oral sucker breadth** | 0.64–0.80 (0.73) | 0.60–0.81 (0.67) | 0.68–0.89 (0.78) | 0.57–0.59 (0.58) | 0.62–0.81 (0.70) | 0.68–0.73 (0.71) | 0.68–0.80 (0.72) | 0.61–0.82 (0.69) | 0.54–0.77 (0.62) | 0.53–0.64 (0.57) | 0.68–0.84 (0.76) | 0.55–0.68 (0.62) | 0.63–0.70 (0.67) | 0.64–0.76 (0.70) | 0.69–0.72 (0.71) | 0.72–1.14 (0.86) | 0.63–0.81 (0.72) | 0.69–1.03 (0.80) | 0.62–0.94 (0.77) | 0.91–1.01 (0.97) |
| **Left caecum length** | 308–647 (515) | 361–867 (597) | 280–403 (348) | 723–816 (755) | 376–519 (461) | 342–406 (374) | 411–582 (499) | 434–657 (495) | 637–1074 (863) | 468–842 (622) | 306–461 (377) | 346–843 (568) | 427–631 (507) | 394–545 (451) | 316–334 (325) | 370–754 (547) | 390–742 (552) | 336–962 (510) | 756–984 (871) | 549–559 (554) |
| **Right caecum length** | 373–649 (524) | 425–927 (602) | 273–424 (345) | 597–763 (706) | 370–565 (482) | 378–383 (381) | 388–650 (513) | 386–656 (502) | 637–1117 (887) | 475–869 (640) | 285–469 (381) | 376–853 (576) | 436–612 (514) | 386–571 (455) | 300–304 (302) | 375–746 (552) | 418–712 (552) | 372–988 (517) | 802–1036 (883) | 449–523 (486) |
| **True caecal space as % body length** | 44.1–68.1 (54.1) | 55.4–64.4 (58.8) | 46.0–61.3 (51.7) | 56.2–57.5 (56.7) | 49.4–62.0 (56.3) | 56.5–63.8 (60.2) | 54.6–68.0 (59.4) | 49.8–67.1 (56.7) | 48.1–68.6 (60.6) | 50.2–66.4 (59.1) | 44.0–61.2 (52.9) | 48.9–58.1 (54.5) | 48.7–57.9 (54.2) | 48.1–63.5 (55.5) | 45.4–48.2 (46.8) | 54.9–70.3 (62.2) | 52.9–60.3 (56.4) | 45.9–57.7 (52.5) | 53.9–68.1 (61.3) | 53.8–57.5 (55.6) |
| **Left testis length** | 107–217 (164) | 124–237 (172) | 99–120 (107) | 151–190 (170) | 78–178 (139) | 77–82 (80) | 126–169 (148) | 105–163 (129) | 177–221 (203) | 102–210 (143) | 83–135 (102) | 72–200 (132) | 123–177 (152) | 91–119 (110) | 89–113 (101) | 83–264 (132) | 104–171 (129) | 67–260 (117) | 134–194 (167) | 131–203 (170) |
| **Left testis length as % body length** | 13.8–19.7 (16.2) | 14.2–19.6 (16.6) | 13.5–16.7 (15.3) | 13.1–13.8 (13.4) | 12.4–19.1 (15.5) | 10.7–13.7 (12.2) | 15.4–18.7 (17.0) | 12.1–18.7 (14.4) | 11.9–17.2 (13.9) | 10.7–16.0 (13.0) | 11.5–17.7 (13.8) | 10.1–14.4 (12.3) | 14.2–17.4 (15.7) | 12.0–14.2 (13.1) | 13.6–15.4 (14.5) | 11.1–19.2 (14.1) | 10.5–18.2 (13.4) | 9.1–16.3 (10.8) | 9.0–13.2 (11.7) | 13.7–19.5 (16.6) |
| **Left testis breadth** | 96–181 (145) | 116–186 (145) | 76–103 (88) | 135–179 (159) | 72–162 (117) | 68–83 (76) | 106–144 (123) | 106–139 (126) | 155–197 (172) | 95–178 (127) | 74–116 (96) | 68–179 (116) | 119–172 (143) | 90–116 (106) | 86–105 (96) | 68–192 (106) | 83–136 (110) | 52–207 (93) | 106–155 (136) | 118–158 (144) |
| **Left testis breadth as % body breadth** | 19.0–24.0 (21.7) | 20.1–28.5 (23.3) | 17.2–23.8 (20.7) | 22.7–27.0 (24.8) | 17.6–25.2 (20.2) | 15.7–21.1 (18.4) | 19.6–24.4 (21.2) | 19.1–26.2 (23.1) | 20.0–26.1 (22.5) | 16.4–25.1 (20.3) | 17.8–24.1 (20.7) | 16.6–22.7 (19.4) | 19.9–22.6 (21.1) | 17.8–21.3 (19.8) | 20.5–22.9 (21.7) | 15.5–30.3 (23.4) | 18.0–26.8 (22.5) | 15.6–26.7 (21.0) | 18.8–26.6 (23.0) | 20.7–24.8 (23.4) |
| **Right testis length** | 91–211 (165) | 124–238 (179) | 99–136 (112) | 155–188 (176) | 80–168 (133) | 81–82 (82) | 119–175 (145) | 110–141 (126) | 160–218 (195) | 96–208 (142) | 79–114 (100) | 83–221 (136) | 131–189 (160) | 92–128 (110) | 87–105 (96) | 80–203 (134) | 95–166 (126) | 65–262 (123) | 139–192 (165) | 141–230 (182) |
| **Right testis length as % body length** | 13.4–19.2 (16.1) | 14.5–21.1 (17.3) | 14.6–17.2 (15.9) | 12.3–14.2 (13.4) | 12.7–17.2 (15.0) | 11.3–13.7 (12.5) | 15.6–18.3 (16.7) | 10.9–16.2 (14.1) | 11.9–15.3 (13.4) | 10.7–15.8 (12.9) | 10.9–15.6 (13.5) | 10.7–14.4 (12.6) | 14.7–20.0 (16.6) | 10.9–15.4 (13.2) | 13.3–14.3 (13.8) | 10.7–19.6 (14.5) | 11.7–14.7 (12.8) | 8.7–16.4 (11.0) | 9.6–13.5 (11.4) | 14.8–22.1 (18.4) |
| **Right testis breadth** | 100–174 (144) | 107–196 (152) | 92–115 (102) | 148–179 (158) | 72–140 (117) | 66–79 (73) | 89–159 (131) | 105–130 (118) | 139–193 (170) | 94–163 (129) | 75–119 (97) | 71–197 (118) | 127–165 (150) | 91–126 (102) | 79–115 (97) | 67–197 (108) | 79–136 (103) | 47–202 (96) | 116–152 (132) | 106–180 (151) |
| **Right testis breadth as % body breadth** | 18.6–23.7 (21.5) | 19.2–29.6 (24.5) | 22.1–26.1 (23.8) | 23.5–25.3 (24.7) | 17.6–22.7 (20.3) | 15.2–20.1 (17.7) | 17.4–26.6 (22.5) | 16.9–23.9 (21.8) | 17.8–29.2 (22.3) | 16.9–23.9 (20.7) | 18.2–26.2 (21.0) | 17.4–23.3 (19.7) | 20.6–24.5 (22.2) | 17.9–21.4 (19.2) | 21.1–22.4 (21.7) | 16.3–34.3 (23.9) | 16.9–24.3 (20.9) | 14.1–29.8 (21.2) | 19.2–24.9 (22.4) | 18.6–28.1 (24.4) |
| **Area left testis** | 33.8–105.7 (77.0) | 45.2–138.4 (80.8) | 24.1–34.9 (29.7) | 69.9–106.8 (85.3) | 17.6–90.5 (53.1) | 16.4–21.4 (18.9) | 41.9–75.5 (57.7) | 34.9–71.1 (51.7) | 89.5–121.9 (109.5) | 30.4–104.2 (58.7) | 19.3–49.2 (31.0) | 15.4–112.4 (51.0) | 46.0–94.0 (69.3) | 26.0–42.6 (36.8) | 24.0–37.3 (30.6) | 17.9–159.2 (47.8) | 27.4–66.6 (45.1) | 11.2–160.0 (40.3) | 44.6–86.1 (71.8) | 48.5–100.7 (78.5) |
| **Area right testis** | 28.6–107.1 (77.1) | 41.7–144.6 (88.1) | 28.6–49.1 (36.0) | 72.0–103.4 (87.7) | 18.1–68.1 (50.7) | 16.8–20.3 (18.6) | 33.3–84.6 (60.8) | 36.3–57.6 (46.9) | 73.5–132.1 (104.9) | 28.9–99.9 (59.1) | 18.8–41.8 (30.5) | 18.9–136.7 (55.4) | 52.2–97.9 (75.8) | 26.6–43.4 (35.5) | 21.6–37.9 (29.7) | 17.3–125.6 (48.8) | 23.6–66.2 (41.8) | 10.2–162.3 (43.5) | 50.6–91.6 (68.9) | 46.9–130.0 (89.2) |
| **Area left testis as % area ventral sucker** | 38.4–75.2 (53.1) | 37.5–70.1 (50.8) | 40.6–53.4 (45.3) | 40.9–59.2 (50.1) | 34.0–81.7 (49.8) | 26.7–43.9 (35.3) | 48.5–66.5 (58.1) | 40.1–75.3 (53.9) | 36.8–66.1 (49.7) | 23.6–54.7 (34.7) | 35.7–58.6 (48.1) | 24.1–49.3 (36.6) | 39.9–64.2 (53.2) | 42.0–53.2 (48.0) | 44.3–47.3 (45.8) | 21.1–86.2 (48.9) | 27.6–58.0 (40.1) | 18.1–46.9 (29.8) | 23.6–48.1 (40.1) | 59.1–109.8 (80.2) |
| **Area right restis as % area ventral sucker** | 43.1–79.6 (52.6) | 28.8–76.8 (56.9) | 45.3–69.0 (54.8) | 42.1–61.7 (51.7) | 34.9–59.1 (47.6) | 27.3–41.8 (34.5) | 39.6–78.5 (60.7) | 38.3–60.9 (49.2) | 28.3–64.6 (47.3) | 24.0–50.0 (35.1) | 31.5–60.9 (48.0) | 28.6–54.4 (38.3) | 48.4–78.2 (58.9) | 40.3–55.2 (46.4) | 39.7–48.1 (43.9) | 23.3–89.1 (50.5) | 27.8–48.7 (35.7) | 15.8–51.3 (30.8) | 29.8–51.1 (38.3) | 57.1–141.8 (91.5) |
| **Anterior distance left testis** | 275–506 (393) | 183–476 (310) | 170–331 (228) | 339–399 (374) | 224–321 (277) | 199–230 (215) | 241–308 (274) | 230–348 (275) | 341–489 (428) | 279–460 (341) | 200–275 (244) | 228–477 (320) | 255–386 (310) | 235–325 (265) | 212–241 (227) | 261–396 (329) | 236–516 (362) | 250–641 (357) | 415–498 (465) | 251–298 (275) |
| **Anterior distance right testis** | 275–533 (394) | 158–449 (310) | 184–268 (216) | 350–384 (367) | 226–306 (275) | 203–244 (224) | 247–301 (279) | 250–364 (281) | 353–507 (440) | 263–442 (349) | 204–286 (250) | 223–473 (331) | 254–372 (312) | 223–325 (272) | 214–234 (224) | 258–528 (343) | 250–740 (474) | 253–604 (361) | 425–532 (471) | 253–299 (278) |
| **True pre-testicular space** | 275–506 (385) | 158–449 (304) | 170–268 (212) | 339–384 (368) | 224–306 (269) | 199–230 (215) | 241–301 (271) | 230–348 (273) | 341–484 (424) | 263–435 (337) | 200–270 (241) | 223–451 (314) | 254–372 (306) | 223–325 (260) | 212–234 (223) | 258–396 (327) | 236–482 (354) | 250–604 (356) | 415–494 (458) | 251–298 (275) |
| **Pre-test space as % body length** | 34.2–46.3 (38.1) | 17.5–38.7 (29.3) | 27.9–33.8 (30.1) | 26.9–29.7 (28.1) | 26.9–35.6 (30.8) | 32.0–33.2 (32.6) | 28.3–34.2 (31.2) | 27.6–32.1 (30.2) | 26.3–31.1 (28.8) | 26.7–34.2 (30.8) | 30.2–36.0 (32.8) | 27.1–33.0 (29.7) | 30.2–33.5 (31.5) | 28.7–33.5 (30.9) | 31.8–32.4 (32.1) | 33.4–42.3 (36.5) | 32.6–41.8 (35.3) | 28.7–36.9 (33.5) | 30.4–35.0 (32.5) | 26.3–26.4 (26.4) |
| **Posterior distance left testis** | 331–730 (545) | 417–908 (635) | 372–443 (415) | 836–875 (853) | 372–605 (521) | 351–403 (377) | 396–562 (494) | 425–725 (517) | 749–1078 (885) | 528–784 (661) | 362–496 (416) | 434–942 (652) | 467–667 (559) | 431–599 (503) | 381–421 (401) | 370–627 (485) | 262–765 (452) | 360–921 (563) | 796–1003 (884) | 632–639 (636) |
| **Posterior distance right testis** | 363–723 (528) | 434–909 (634) | 373–453 (422) | 832–898 (858) | 368–618 (524) | 356–407 (382) | 438–549 (504) | 484–715 (538) | 739–1075 (904) | 535–780 (665) | 359–493 (417) | 420–938 (647) | 469–658 (559) | 423–586 (509) | 390–451 (421) | 352–648 (493) | 427–710 (562) | 389–1010 (581) | 825–1009 (899) | 587–605 (596) |
| **True post-testicular space** | 331–723 (522) | 417–908 (625) | 372–443 (412) | 832–875 (851) | 368–605 (513) | 351–403 (377) | 396–549 (489) | 425–715 (512) | 739–1075 (880) | 528–765 (652) | 359–493 (413) | 420–938 (642) | 467–658 (548) | 423–586 (500) | 381–421 (401) | 352–627 (478) | 262–700 (442) | 360–1010 (573) | 796–1003 (884) | 587–605 (596) |
| **Post-test space as % body length** | 44.2–56.7 (51.1) | 53.1–68.1 (59.7) | 53.2–61.5 (58.9) | 63.5–67.0 (64.9) | 55.5–61.2 (58.0) | 56.1–58.5 (57.3) | 51.6–61.3 (56.1) | 48.8–59.6 (56.4) | 55.8–63.9 (59.8) | 53.6–63.9 (59.5) | 49.8–60.3 (56.1) | 55.8–62.8 (60.2) | 54.7–58.5 (56.4) | 57.1–62.7 (59.5) | 57.3–58.2 (57.7) | 40.3–56.7 (53.3) | 33.8–57.1 (44.9) | 52.0–63.0 (57.5) | 59.2–63.9 (61.8) | 58.2–61.5 (59.8) |
| **Ovary length** | 64–147 (97) | 85–158 (114) | 52–71 (62) | 113–134 (122) | 45–106 (80) | 52–74 (63) | 73–121 (92) | 71–116 (90) | 100–159 (134) | 75–135 (99) | 38–90 (66) | 65–163 (95) | 57–111 (89) | 48–86 (67) | 63–64 (64) | 50–120 (81) | 72–164 (97) | 49–245 (89) | 131–181 (150) | 114–128 (123) |
| **Ovary breadth** | 49–183 (98) | 74–144 (108) | 50–74 (60) | 103–123 (113) | 56–88 (75) | 48–66 (57) | 69–137 (89) | 72–87 (81) | 79–151 (127) | 68–129 (101) | 37–79 (59) | 51–170 (84) | 60–101 (81) | 47–77 (60) | 57–58 (58) | 38–113 (70) | 57–78 (66) | 39–152 (67) | 69–120 (91) | 61–74 (67) |
| **Ovary breadth / length** | 0.77–1.39 (1.00) | 0.69–1.16 (0.95) | 0.74–1.19 (0.99) | 0.82–1.01 (0.93) | 0.73–1.24 (0.98) | 0.89–0.92 (0.91) | 0.84–1.13 (0.97) | 0.75–1.05 (0.91) | 0.78–1.11 (0.95) | 0.81–1.22 (1.03) | 0.60–1.14 (0.91) | 0.70–1.04 (0.89) | 0.82–1.05 (0.92) | 0.73–1.00 (0.90) | 0.89–0.92 (0.91) | 0.63–1.38 (0.87) | 0.37–0.98 (0.73) | 0.54–1.14 (0.78) | 0.46–0.85 (0.61) | 0.52–0.58 (0.55) |
| **Ovary length as % body length** | 7.80–11.57 (9.54) | 8.18–13.64 (11.11) | 7.51–10.58 (8.88) | 8.35–10.36 (9.22) | 6.75–11.91 (8.93) | 8.67–10.31 (9.49) | 8.35–12.66 (10.52) | 8.69–11.04 (9.96) | 7.45–11.45 (9.16) | 6.27–11.23 (9.09) | 5.70–12.80 (8.99) | 6.21–11.72 (8.84) | 5.54–11.85 (9.31) | 5.35–11.40 (8.05) | 8.57–9.77 (9.17) | 6.14–12.63 (9.20) | 6.88–14.24 (9.70) | 6.10–10.03 (7.83) | 10.02–11.50 (10.68) | 5.87–7.02 (6.44) |
| **Hermaphroditic sac length** | 67–137 (108) | 92–158 (122) | 76–92 (81) | 135–154 (143) | 63–128 (109) | 77–91 (84) | 107–134 (117) | 94–143 (109) | 126–191 (149) | 71–178 (112) | 83–123 (106) | 68–174 (118) | 111–165 (136) | 98–130 (116) | 108–118 (113) | 78–188 (137) | 88–187 (134) | 79–275 (135) | 116–198 (149) | 97–121 (107) |
| **Hermaphroditic sac breadth** | 57–133 (101) | 81–142 (112) | 68–101 (86) | 113–128 (122) | 56–119 (100) | 83–90 (87) | 85–114 (98) | 87–116 (97) | 114–150 (130) | 77–147 (104) | 72–116 (89) | 60–140 (97) | 91–151 (119) | 83–103 (93) | 82–99 (91) | 74–150 (112) | 71–179 (118) | 61–246 (114) | 104–163 (137) | 91–105 (97) |
| **Herm sac breadth / length** | 0.82–1.04 (0.94) | 0.75–1.07 (0.92) | 0.86–1.24 (1.07) | 0.83–0.89 (0.85) | 0.73–1.17 (0.93) | 0.91–1.17 (1.04) | 0.79–0.91 (0.84) | 0.81–1.02 (0.89) | 0.72–0.97 (0.88) | 0.75–1.14 (0.95) | 0.72–1.13 (0.85) | 0.73–0.98 (0.82) | 0.71–1.08 (0.88) | 0.70–0.91 (0.80) | 0.69–0.92 (0.81) | 0.62–1.20 (0.83) | 0.66–1.03 (0.87) | 0.57–1.26 (0.84) | 0.69–1.22 (0.93) | 0.87–0.98 (0.91) |
| **Herm sac length as % body length** | 9.49–12.47 (10.64) | 9.64–14.39 (11.92) | 10.10–13.29 (11.65) | 10.70–11.18 (10.98) | 9.82–14.37 (12.20) | 12.67–12.83 (12.75) | 12.10–14.34 (13.41) | 10.79–13.68 (12.11) | 8.54–11.37 (10.14) | 7.49–13.56 (10.14) | 11.32–16.44 (14.39) | 9.34–13.12 (11.17) | 12.44–16.65 (14.08) | 11.40–17.58 (13.98) | 14.69–18.02 (16.35) | 11.42–18.35 (15.19) | 12.22–15.19 (13.44) | 9.17–17.22 (13.02) | 7.63–13.94 (10.30) | 10.00–12.67 (11.34) |
| **Anterior distance hermaphroditic sac** | 192–327 (261) | 78–306 (209) | 37–162 (116) | 206–253 (232) | 131–211 (180) | 125–130 (128) | 156–183 (168) | 149–233 (176) | 211–302 (267) | 174–287 (217) | 129–185 (158) | 132–281 (201) | 150–255 (196) | 142–200 (164) | 133–152 (143) | 174–256 (218) | 169–342 (233) | 144–425 (243) | 270–320 (290) | 191–281 (250) |
| **Posterior distance hermaphroditic sac** | 397–888 (649) | 179–1042 (638) | 137–563 (382) | 949–995 (971) | 430–673 (583) | 389–466 (428) | 470–650 (567) | 546–819 (605) | 833–1178 (1027) | 606–916 (754) | 396–530 (457) | 470–1112 (734) | 537–750 (626) | 452–672 (556) | 388–478 (433) | 405–760 (558) | 441–824 (631) | 433–1189 (641) | 923–1175 (1037) | 538–596 (567) |
| **Pre-herm sac space as % body length** | 20.9–31.7 (25.9) | 8.6–28.3 (19.9) | 6.1–22.3 (16.3) | 16.3–18.6 (17.7) | 17.9–21.7 (20.4) | 18.1–20.8 (19.5) | 17.3–21.1 (19.4) | 17.9–20.4 (19.5) | 15.7–21.4 (18.1) | 17.6–23.0 (19.8) | 18.3–25.3 (21.6) | 14.8–23.7 (19.1) | 17.8–23.0 (20.1) | 18.0–21.9 (19.5) | 20.3–20.7 (20.5) | 17.2–30.6 (24.5) | 21.8–25.8 (23.5) | 20.8–25.7 (23.0) | 18.5–21.8 (20.4) | 20.0–26.8 (23.4) |
| **Post-herm sac space as % body length** | 58.5–69.2 (63.2) | 17.8–81.3 (59.5) | 19.8–71.8 (53.9) | 72.2–76.7 (74.1) | 58.4–68.8 (65.9) | 64.8–64.9 (64.9) | 62.4–68.0 (65.0) | 65.0–68.3 (66.8) | 65.6–73.0 (69.8) | 62.6–71.9 (68.8) | 57.7–67.0 (62.1) | 63.2–72.5 (68.7) | 61.0–67.0 (64.6) | 62.1–69.2 (66.0) | 59.2–65.0 (62.1) | 55.5–66.8 (62.1) | 60.0–67.7 (63.9) | 58.4–70.4 (64.3) | 68.9–77.7 (72.3) | 56.3–57.3 (56.8) |
| **Left margin distance herm sac** | 179–277 (245) | 143–294 (207) | 53–199 (118) | 210–251 (231) | 140–227 (187) | 148–163 (156) | 162–227 (194) | 156–210 (184) | 208–312 (251) | 140–232 (198) | 120–199 (155) | 117–300 (194) | 194–265 (226) | 142–203 (181) | 143–183 (163) | 113–240 (157) | 126–191 (167) | 95–304 (151) | 177–244 (210) | 202–226 (218) |
| **Right margin distance herm sac** | 178–273 (240) | 173–704 (317) | 37–528 (206) | 194–256 (227) | 150–217 (197) | 135–145 (140) | 145–223 (186) | 161–274 (189) | 203–297 (247) | 161–280 (201) | 125–177 (155) | 118–315 (190) | 203–289 (240) | 166–210 (183) | 134–168 (151) | 117–219 (160) | 133–229 (184) | 91–307 (149) | 170–227 (203) | 191–272 (237) |
| **Seminal vesicle length** | 20–59 (44) | 34–80 (54) | 26–46 (33) | 40–66 (51) | 25–79 (51) | 29–34 (32) | 39–51 (45) | 33–50 (42) | 38–83 (51) | 20–60 (41) | 29–49 (37) | 31–82 (46) | 31–62 (49) | 32–48 (40) | 40–47 (44) | 36–74 (51) | 32–89 (56) | 32–89 (55) | 36–83 (54) | 30–53 (41) |
| **Seminal vesicle breadth** | 31–52 (42) | 39–75 (56) | 23–59 (36) | 37–69 (56) | 33–73 (57) | 37–47 (42) | 38–73 (55) | 46–66 (52) | 36–74 (56) | 30–81 (47) | 27–59 (41) | 33–66 (51) | 42–83 (55) | 38–59 (47) | 39–39 (39) | 39–61 (48) | 36–65 (51) | 32–111 (56) | 34–76 (49) | 29–46 (39) |
| **Genital pore left margin distance** | 199–324 (277) | 144–311 (222) | 47–230 (138) | 227–296 (261) | 151–268 (214) | 139–151 (145) | 177–243 (211) | 177–231 (205) | 240–327 (279) | 162–263 (218) | 124–225 (166) | 138–308 (221) | 210–330 (256) | 141–223 (192) | 150–210 (180) | 135–292 (183) | 143–263 (208) | 128–359 (183) | 231–283 (251) | 203–275 (232) |
| **Genital pore right margin distance** | 195–339 (273) | 179–357 (246) | 70–176 (140) | 227–276 (256) | 156–241 (214) | 146–159 (153) | 166–256 (209) | 170–300 (211) | 211–351 (271) | 174–286 (227) | 122–207 (171) | 126–336 (204) | 203–311 (253) | 186–233 (203) | 152–175 (164) | 143–254 (185) | 153–275 (218) | 110–366 (184) | 224–270 (248) | 216–287 (259) |
| **Genital pore anterior distance** | 205–387 (282) | 113–343 (236) | 41–163 (108) | 212–265 (245) | 140–221 (191) | 124–131 (128) | 167–209 (184) | 166–261 (189) | 201–312 (274) | 188–304 (228) | 130–187 (162) | 150–286 (208) | 161–272 (208) | 145–195 (166) | 140–157 (149) | 177–289 (235) | 187–405 (257) | 165–459 (260) | 287–359 (312) | 231–314 (276) |
| **Genital pore posterior distance** | 447–940 (716) | 530–1138 (813) | 258–625 (532) | 1065–1132 (1088) | 455–730 (655) | 435–568 (502) | 559–729 (650) | 609–936 (687) | 978–1354 (1151) | 659–1037 (840) | 480–617 (540) | 526–1219 (823) | 622–850 (730) | 546–758 (642) | 487–546 (517) | 460–876 (664) | 492–950 (726) | 518–1314 (741) | 1036–1266 (1157) | 699–725 (712) |
| **Pre-genital pore space as % body length** | 22.4–30.5 (27.9) | 12.5–31.0 (22.8) | 6.7–21.8 (15.1) | 16.8–20.0 (18.7) | 19.5–23.0 (21.6) | 17.3–21.8 (19.6) | 18.9–23.2 (21.2) | 19.9–21.8 (20.9) | 15.0–22.2 (18.6) | 18.4–23.3 (20.8) | 19.1–26.5 (22.1) | 15.3–24.6 (19.9) | 19.1–24.5 (21.3) | 18.2–21.4 (19.8) | 21.4–21.4 (21.4) | 23.4–33.0 (26.3) | 23.1–30.6 (25.9) | 22.7–27.1 (24.5) | 19.2–23.2 (21.9) | 24.2–27.2 (25.7) |
| **Post-genital pore space as % body length** | 65.8–75.3 (70.0) | 67.5–88.5 (77.7) | 42.4–86.8 (75.0) | 82.1–84.5 (83.0) | 72.2–76.5 (73.9) | 72.5–79.1 (75.8) | 72.3–76.3 (74.6) | 74.5–78.0 (75.7) | 74.6–81.6 (78.3) | 73.9–80.7 (76.6) | 70.0–76.7 (73.5) | 71.7–82.2 (77.0) | 72.5–76.4 (75.3) | 74.5–78.1 (76.4) | 74.3–74.4 (74.3) | 64.7–78.0 (73.8) | 67.2–77.9 (73.4) | 70.7–78.6 (74.4) | 78.0–83.3 (80.7) | 69.7–73.2 (71.5) |
| **Egg1 length** | 81–99 (90) | 78–98 (90) | 83–102 (94) | 93–103 (96) | 84–96 (89) | 74–85 (80) | 85–100 (90) | 81–96 (90) | 79–102 (91) | 84–99 (90) | 82–95 (89) | 80–98 (90) | 87–100 (93) | 80–92 (87) | 78–90 (84) | 55–94 (79) | 65–85 (79) | 68–88 (79) | 89–102 (92) | 84–91 (88) |
| **Egg1 breadth** | 41–48 (45) | 39–54 (46) | 41–54 (46) | 50–74 (62) | 49–58 (53) | 50–53 (52) | 51–62 (55) | 46–61 (53) | 52–59 (54) | 50–70 (57) | 43–58 (52) | 44–61 (54) | 49–57 (53) | 53–59 (55) | 55–67 (61) | 30–47 (40) | 40–52 (44) | 33–53 (42) | 40–55 (47) | 39–47 (44) |
| **Egg2 length** | 74–94 (90) | 66–103 (87) | 87–96 (92) | 90–99 (94) | 77–90 (86) | 81–81 (81) | 87–96 (91) | 79–94 (87) | 83–99 (92) | 78–95 (89) | 74–97 (86) | 70–98 (89) | 81–103 (91) | 82–98 (90) | 86–87 (87) | 63–90 (81) | 73–87 (78) | 69–91 (80) | 83–98 (89) | 84–93 (90) |
| **Egg2 breadth** | 41–54 (47) | 44–62 (50) | 44–56 (49) | 56–60 (57) | 50–62 (55) | 46–46 (46) | 52–62 (56) | 52–59 (56) | 46–61 (55) | 50–68 (56) | 47–65 (55) | 44–58 (54) | 50–55 (53) | 46–57 (53) | 53–62 (58) | 37–47 (42) | 36–54 (45) | 34–58 (43) | 39–55 (47) | 42–50 (45) |
| **Egg3 length** | 84–94 (89) | 81–101 (92) | 85–93 (89) | 94–107 (99) | 77–88 (85) | – () | 80–96 (85) | 77–96 (88) | 86–99 (90) | 70–95 (87) | 69–94 (84) | 79–106 (91) | 82–102 (91) | 79–95 (87) | 63–87 (75) | 59–91 (80) | 70–86 (78) | 68–86 (77) | 76–93 (85) | 81–97 (90) |
| **Egg3 breadth** | 38–54 (45) | 41–59 (49) | 42–58 (48) | 53–55 (54) | 41–62 (53) | – () | 53–59 (56) | 47–59 (52) | 52–58 (55) | 50–62 (56) | 45–63 (56) | 44–60 (53) | 49–66 (54) | 48–57 (53) | 44–61 (53) | 32–45 (41) | 35–46 (40) | 33–55 (42) | 44–55 (49) | 39–50 (44) |
| **No. of eggs** | 0–70 (24) | 2–39 (22) | 0–8 (6) | 31–60 (49) | 0–25 (16) | 1–3 (2) | 3–17 (11) | 7–30 (12) | 16–54 (33) | 5–45 (23) | 2–10 (7) | 3–44 (16) | 9–28 (18) | 7–18 (11) | 5–7 (6) | 0–53 (13) | 4–15 (8) | 0–77 (10) | 14–32 (20) | 39–60 (50) |
| **Vitellarium anterior extent distance** | 185–365 (258) | 152–342 (251) | 110–267 (172) | 252–413 (319) | 119–206 (141) | 159–169 (164) | 155–192 (175) | 141–262 (176) | 255–373 (334) | 213–352 (280) | 151–350 (198) | 202–317 (249) | 124–223 (179) | 142–224 (190) | 171–197 (184) | 191–314 (251) | 137–459 (278) | 148–496 (296) | 372–666 (517) | 129–182 (148) |
| **Vitellarium posterior extent distance** | 86–361 (205) | 131–366 (229) | 146–213 (189) | 217–276 (248) | 158–270 (211) | 101–187 (144) | 123–194 (151) | 145–225 (181) | 184–318 (239) | 133–236 (180) | 104–202 (145) | 138–304 (206) | 120–246 (195) | 40–217 (161) | 155–175 (165) | 93–207 (162) | 126–313 (204) | 167–339 (233) | 258–412 (300) | 210–275 (243) |
| **Pre-vitellar space as % body length** | 20.1–33.4 (25.5) | 16.9–39.9 (24.6) | 18.1–33.7 (24.1) | 20.0–30.0 (24.1) | 12.0–23.1 (15.2) | 23.5–26.5 (25.0) | 18.2–22.0 (20.2) | 16.6–21.8 (19.3) | 19.9–27.8 (22.8) | 20.2–30.5 (25.6) | 18.5–45.9 (27.0) | 16.7–29.9 (24.3) | 12.1–21.1 (18.4) | 16.9–25.9 (22.7) | 26.1–26.8 (26.5) | 23.5–35.7 (28.3) | 17.6–34.6 (27.1) | 15.5–48.4 (29.8) | 29.3–46.6 (37.6) | 13.5–17.5 (15.5) |
| **Post-vitellar space as % body length** | 12.6–26.8 (19.5) | 16.6–25.4 (21.6) | 24.0–29.5 (26.9) | 17.2–21.3 (18.9) | 17.0–27.3 (22.4) | 16.8–26.0 (21.4) | 13.6–20.8 (17.4) | 17.7–23.5 (20.1) | 12.6–24.0 (16.4) | 11.7–21.0 (16.5) | 15.5–24.7 (19.6) | 15.9–21.7 (19.4) | 14.2–24.0 (20.0) | 5.5–24.2 (18.9) | 23.7–23.8 (23.7) | 12.5–24.0 (18.1) | 15.4–25.7 (20.6) | 14.9–33.8 (23.8) | 18.9–26.2 (21.1) | 22.0–26.4 (24.2) |
| **Vitellar space as % body length** | 45.7–64.3 (55.0) | 42.5–60.6 (53.8) | 39.4–58.0 (49.0) | 51.9–62.8 (57.0) | 55.3–69.2 (62.3) | 50.4–56.7 (53.5) | 59.3–66.6 (62.4) | 59.4–62.9 (60.6) | 48.2–67.5 (60.8) | 50.9–66.0 (57.8) | 33.6–61.1 (53.4) | 48.7–64.4 (56.3) | 58.6–65.4 (61.6) | 54.3–69.6 (58.4) | 49.4–50.2 (49.8) | 46.8–61.2 (53.6) | 44.8–62.1 (52.3) | 26.9–64.0 (46.4) | 31.5–48.4 (41.3) | 56.1–64.5 (60.3) |
